# The role of health literacy in cancer care: A mixed studies systematic review

**DOI:** 10.1371/journal.pone.0259815

**Published:** 2021-11-12

**Authors:** Chloe E. Holden, Sally Wheelwright, Amélie Harle, Richard Wagland

**Affiliations:** 1 Health Sciences, University of Southampton, Southampton, Hampshire, United Kingdom; 2 Dorset Cancer Centre, University Hospitals Dorset NHS Foundation Trust, Poole, Dorset, United Kingdom; University College London, UNITED KINGDOM

## Abstract

**Background:**

Patients diagnosed with cancer face many challenges and need a good understanding of their diagnosis and proposed treatments to make informed decisions about their care. Health literacy plays an important role in this and low health literacy has been associated with poorer outcomes. The aims of this review are to identify which outcomes relate to health literacy in patients with cancer, and to combine this through a mixed studies approach with the patient experience as described through qualitative studies.

**Methods:**

Four electronic databases were searched in January 2021 to identify records relating to health literacy and patients with cancer. Records were independently screened then assessed for inclusion by two reviewers according to the following criteria: patients aged ≥18 years with cancer, English language publication AND health literacy measured with validated tool and measured outcome associated with health literacy OR qualitative study exploring the role of health literacy as patients make decisions about health. Quality was independently assessed by two reviewers. A narrative synthesis was performed, and findings integrated through concept mapping. This systematic review was registered with PROSPERO, entry CRD42020166454.

**Results:**

4441 records were retrieved. Following de-duplication, 2496 titles and abstracts were screened and full texts of 405 papers were reviewed for eligibility. 66 papers relating to 60 studies met the eligibility criteria. Lower health literacy was associated with greater difficulties understanding and processing cancer related information, poorer quality of life and poorer experience of care. Personal and situational influences contributed to how participants processed information and reached decisions about their care.

**Conclusion:**

This review highlights the important role of health literacy for patients with cancer. Outcomes are poorer for those who experience difficulties with health literacy. Further efforts should be made to facilitate understanding, develop health literacy and support patients to become more involved in their care.

## Introduction

Patients with cancer are expected to understand complicated information about their diagnosis and management. They must learn a new language of health terminology, provide consent for treatments and procedures, turn up at the right time and place for their appointments and seek help in an appropriate way and in a timely manner. Health literacy is integral to this.

Health literacy has been defined as “the combination of personal competencies and situational resources needed for people to access, understand, appraise and use information and services to make decisions about health. It includes the capacity to communicate, assert and act upon these decisions” [1]. Two distinct views of health literacy, as either a ‘risk’ or an ‘asset’, have been proposed [2]. The ‘risk’ approach is largely associated with work in the clinical domain, exploring the impact of health literacy on individual and health system outcomes. Health literacy in this context is used to describe an individual’s literacy skills, and low health literacy is seen as a risk factor that must be compensated for. In the ‘asset’ approach however, which has developed from work in public health, health literacy is seen as an asset to be built, comprising more than just functional skills and including the development of more advanced social and communication skills as a means of increasing patient empowerment [2].

As a ‘risk’, health literacy is associated with hospitalisation, use of emergency care, uptake of preventative services, ability to understand health information and take medications appropriately, and, in older people, with health status and mortality [3]. Crucially, it is modifiable [4, 5] and improving health literacy is increasingly recognised as a way of improving outcomes, including in Europe’s Beating Cancer Plan [6].

In addition to relationships with health outcomes, health literacy is a prerequisite for shared and informed decision making [7, 8] and has close ties with person-centred care, which aims to support patients to develop their knowledge, skills and confidence to participate in a partnership with their healthcare provider [9]. Edwards et al.’s Health Literacy Pathway Model considers health literacy from the ‘asset’ perspective, and portrays the development of health literacy as a process over time, influenced by personal, emotional and facilitating factors, leading towards active involvement in consultations and shared decision making [8]. Such involvement is particularly important in cancer care, where patients are often faced with preference-sensitive decisions, and these closely related concepts are therefore very relevant to this setting.

This paper aims to provide an up to date overview of the literature, enabling us to understand the clinical relevance of health literacy in cancer care more broadly than existing reviews focussing on self-management behaviours [10], limited to studies from the USA [11] and exploring interventions to improve health literacy [12] have allowed. Given the complexity of the concept and the personal preference-specific nature of decisions made in the oncology setting, a mixed studies approach was chosen. This was to ensure that the patient voice was heard alongside the quantitative findings and to provide further insight into the patient experience than might have been possible through analysis of measured patient reported outcomes. To our knowledge, no prior systematic reviews have sought to bring together such a comprehensive outline of the field in this way. The objectives were: 1) to identify which outcomes relate to health literacy in patients with cancer and 2) to explore the role and consequences of health literacy, reported by qualitative studies, as patients with cancer access, understand, appraise and use information and services to make decisions about health.

## Methods

The review protocol was prospectively registered with the International Prospective Register for Systematic Reviews (PROSPERO), entry number CRD42020166454. Wording of the qualitative objective has been refined since registration.

### Search strategy

Searches were carried out on four electronic databases (MEDLINE, EMBASE, PsycINFO and CINAHL) in January 2021. Publications addressing cancer and health literacy were sought using search terms identified through review of the existing literature, including MeSH terms (neoplasms, health literacy) and keywords (cancer, malignancy, neoplasm, tumour, carcinoma, health literacy and health competence). Specific outcomes were not stipulated due to the anticipated varied nature of the studies. The search strategy was reviewed by an experienced librarian and is shown in S1 File. Visual scanning of reference lists from included studies was undertaken. Citations were managed through Endnote X9 and Microsoft Excel.

Screening of titles and abstracts was undertaken by two independent reviewers, with one screening all papers (CH) and three reviewers screening a third of papers each (AH, RW, SW), with a preference for inclusion if there were disagreements. Following the initial screening process, full texts of the remaining studies were obtained and independently reviewed for eligibility by two authors (CH and AH, RW or SW) according to the following criteria:

**Inclusion**
- Patients aged ≥18 years with malignancy of any site (if mixed group, data able to be separated)
- English language

**AND**
- Quantitative papers:
  - Health literacy assessed with validated tool (concerning general or cancer health literacy, used in its validated form in its entirety)
  - Measured outcomes associated with health literacy

**OR**
- Qualitative papers:
  - Studies exploring the role of health literacy as patients access, understand, appraise and use information and services to make decisions about health

**Exclusion**
- Use of the term ‘health literacy’ but referring to disease specific knowledge only
- Case reports, review papers, conference proceedings, opinion pieces, editorials, letters to the editor, dissertations/theses, book chapters, protocols

At all stages, disagreements were resolved through discussion.

### Data extraction

One reviewer (CH) extracted data from all papers, with independent extraction from eight papers by a second reviewer (RW, SW) to check for accuracy. Data on study characteristics (author, year, country study undertaken, setting, design, aims/objectives, inclusion/exclusion, recruitment procedure, health literacy measure used and how limited health literacy defined), sample (age range, sex ratio, cancer site, stage, number of participants and number of eligible participants if mixed group, proportion limited health literacy according to measure used and by tumour site), outcomes (as reported in individual studies, measures used and effect of health literacy on these) and qualitative methods used, data analysis procedure, key themes and findings and participant quotes were collected.

### Quality appraisal

Quality was assessed using the Mixed Methods Appraisal Tool (MMAT) [13], allowing all study types to be appraised using a single tool for consistency. MMAT scores are given out of a total of 5, with a point scored for each ‘Yes’ answer, and no points awarded for ‘No’ or ‘Can’t tell’ responses. Studies were considered higher quality if they scored 4/5 or 5/5. Quality assessment was carried out by two independent reviewers, with one assessing all papers (CH), and three assessing a third of the papers each (AH, RW, SW). Disagreements were resolved through discussion.

### Data synthesis

Statistical pooling of data was not performed due to the varied study designs, outcomes, health literacy assessment tools and thresholds used to identify participants with lower health literacy. Drawing on guidance developed by Popay et al. [14], a narrative synthesis was undertaken.

After extraction of the data, studies were grouped and tabulated based on the two review objectives. To address the second, qualitative objective, a thematic analysis was performed [14]. The key themes, authors’ descriptions and interpretations, and supporting quotations were extracted from the results sections of the original qualitative papers, alongside relevant contextual data. Using an iterative process, similar themes were then grouped and used to develop meta-themes, drawing on existing definitions and theoretical frameworks [1, 8, 15]. An initial grouping and development of meta-themes was made by CH before being further refined by RW, after which all authors reviewed the primary texts and met to discuss each meta-theme and contributory theme. Any disagreements were resolved through discussion until consensus on the final grouping was reached.

Finally, relationships between studies across both objectives were explored through concept mapping, again drawing on existing models as appropriate [1, 8], and findings integrated.

## Results

**Fig 1:**
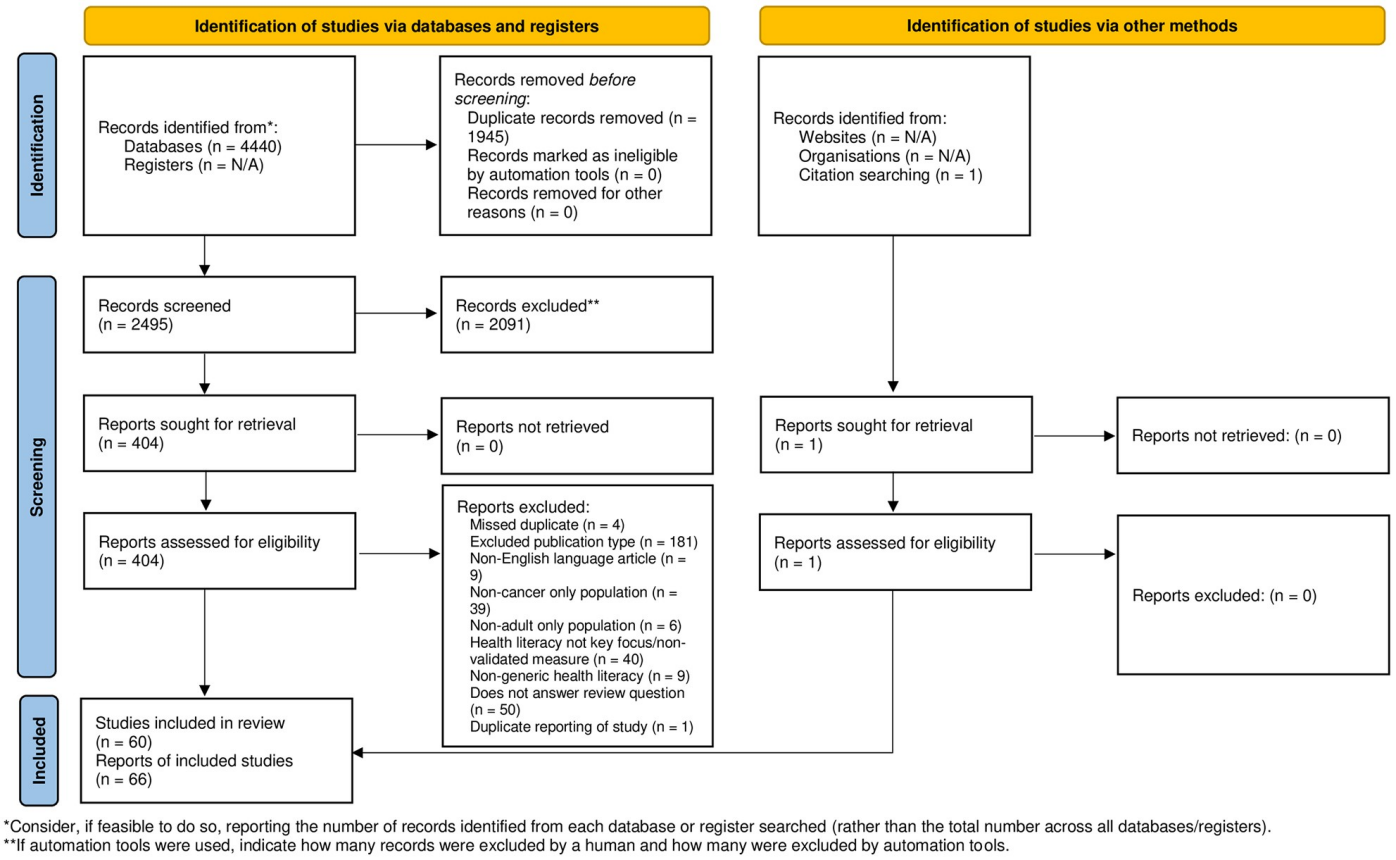
PRISMA diagram showing number of records reviewed at each step of the process. *From:* Page MJ, McKenzie JE, Bossuyt PM, Boutron I, Hoffmann TC, Mulrow CD, et al. The PRISMA 2020 statement: an updated guideline for reporting systematic reviews. BMJ 2021;372:n71. doi: 10.1136/bmj.n71. For more information, visit: http://www.prisma-statement.org/.

4440 records were retrieved from the searches (Fig 1). After removal of duplicates, 2496 titles and abstracts were screened. Full texts of 405 papers were reviewed for eligibility, and 66 papers relating to 60 studies were ultimately selected for inclusion. One additional eligible study was identified through reference list scanning.

### Objective 1: Outcomes relating to health literacy in patients with cancer

Fifty-eight papers relating to 52 studies addressed this objective, of which 49 studies were of quantitative non-randomised design. The majority were conducted in the USA (31/52), and the most common health literacy assessment tools were variations on the Chew screening questions (16/52) and Rapid Estimate of Adult Literacy in Medicine (REALM) (12/52). Breast (N = 12) and prostate cancer (N = 8) were the most studied individual tumour sites, and a further 21 studies included participants with a variety of cancer diagnoses. Thirty-two papers were considered higher quality (MMAT score 4/5 or 5/5). See Table 1 for details of included studies and Table 2 for a summary of the reported associations between health literacy and outcomes. Additional study details can be found in S1 Table. When referring to the significance of associations, the threshold for statistical significance is taken to be p<0.05.

**Table 1:** Included papers reporting associations with health literacy (*N =* 58).

Author, year, location	Participants* Age range % female (n)	Cancer site(s), stage	Outcomes	Outcome measures	Association of health literacy with outcomes	Quality (MMAT score)
Brewer, 2009, USA [16]	133 34–85 years 100%	Breast, stage I-II	Estimating and interpreting recurrence risk Impact of risk results Ease of understanding formats	Measures developed for study	Lower HL: higher and more variable estimates of recurrence risk (p = 0.01), lower ease of understanding (p<0.001)	Higher (5)
Cartwright, 2017, USA [17]	752 NR 50% (377)	Multiple, all stages	Number of admissions Days hospitalised 30-day readmission	Rates from electronic medical records	Lower HL: greater number of inpatient hospital admissions (p = 0.009) and total number of days hospitalised (p = 0.023)	Higher (5)
Hahn, 2010, USA [18]	97 NR 66% (64)	Multiple, stage not reported	Health related quality of life Informed consent comprehension	FACT-G Subset of questions on comprehension based on prior study Other measures developed for study	No significant difference in FACT-G scores	Higher (5)
Husson, 2015, The Netherlands [19]	1643 NR 57% (692)	Colorectal, stage not reported	Health related quality of life Physical activity Mental distress	Questions from European Prospective Investigation into Cancer PA Questionnaire EORTC QLQ-C30 HADS	Lower HL: less likely to meet guidelines for physical activity (p<0.01), negative association with all HRQOL subscales (p<0.01), positive association with mental distress (p<0.01)	Higher (5)
Inglehart, 2016, USA [20]	372 19–89 years 24% (89)	Head and neck, stage not reported	HPV related knowledge Information seeking behaviour	Measures developed for study Utilization and trust in health information based on Health Information National Trends Survey (HINTS)	Higher HL: greater HPV-related knowledge (p<0.01)	Higher (5)
Jiang, 2019, USA [21]	50 41–91 years 40% (20)	Multiple, all stages	Chemotherapy adherence	Medication Event Monitoring System (MEMS^®^)	Higher HL: higher medication adherence (p = 0.03)	Higher (5)
Koay, 2013, Australia [22]	93 27–92 years 17% (16)	Head and neck, lung, stage not reported	Distress	Distress thermometer	Lower HL: increased distress using HeLMS measure (p<0.05) but not using S-TOFHLA measure (p = 0.744)	Higher (5)
Nilsen, 2019, USA [23]	218 NR 23% (51)	Head and neck, stage 0-IV	Quality of life	University of Washington Quality of Life Scale (UWQOL)	Lower HL: lower clinically meaningful social QOL scores (p = 0.013) but not physical QOL scores (p = 0.13)	Higher (5)
Winton, 2016, USA [24]	336 NR NR	Breast, stage 0-IIIA	Type of initial operation for operable breast cancer	Medical record review	Higher HL: greater likelihood of breast reconstruction (non-significant in multivariate analysis, p = 0.06)	Higher (5)
Brewer, 2012, USA [25]	163 36–87 years 100%	Breast, stage I-II	Participant perception of how well results understood	Measures developed for study	Lower HL: lower perceived understanding of test results (p = 0.01)	Higher (4)
Busch, 2015, USA [26]	347 NR 53% (178)	Colorectal, stage I-IV	Receipt of adjuvant chemotherapy Survival	Measures developed for study Social security death index	Higher HL: increased odds of receiving chemotherapy (stage III/IV disease), no association with presentation with early-stage disease (all stages) nor death	Higher (4)
Chan, 2020, Malaysia [27]	345 NR 76% (263)	Multiple, stage I-IV	Preference for patient centred care	Patient Practitioner Orientation Scale (PPOS)	Higher HL: preference for patient centred care (p = 0.001)	Higher (4)
Chang, 2019, Taiwan [28]	120 24–94 years 50% (60)	Multiple, stage not reported	Patient’s assessment of degree of shared decision making	9-item Shared Decision Making Questionnaire (SDM-Q-9)	Higher HL: higher extent to which participants felt involved in shared decision making (p = 0.004)	Higher (4)
Chrischilles, 2019, USA [29]	835 NR 100%	Breast, DCIS-III	Quality of life Upper extremity disability	Disabilities of Arm, Shoulder and Hand Questionnaire short form (QuickDASH) International Classification of Functioning, Disability and Health (ICF) FACT-B	Lower HL: greater disability (p = 0.0062), lower QOL (p = 0.0063)	Higher (4)
Clarke, 2021, Ireland [30]	395 NR 31% (123)	Head and neck, stage I-IV	Health related quality of life Use of self-management behaviours Fear of recurrence	FACT-G FACT-HN Fear of Relapse/Recurrence Scale (FRRS)	Lower HL: lower self-management behaviours and functional wellbeing (p = 0.0220), lower disease specific HRQOL (p = 0.046), higher fear of recurrence (p = 0.040)	Higher (4)
Hendren, 2011, USA [31]	103 NR 90% (93)	Breast and colorectal, stage 0-IV	Patient navigation time	Total time spent with patient and addressing barriers summed and log-transformed to yield a normal distribution	Lower HL: increased navigation time (p = 0.02, non-significant in multivariate analysis)	Higher (4)
İlhan, 2020, Turkey [32]	207 18–83 years 51% (106)	Multiple, stage not reported	Self-care management	Self-Care Management Process in Chronic Illness (SCMP-G).	Lower HL: lower self-care management (p<0.01)	Higher (4)
Lee, 2018, South Korea [33]	80 NR 16% (13)	Lung, stage II-IV (NSCLC), all stages (SCLC)	Quality of life Self-care behaviours	Self-care behaviours measured using previously developed unpublished tool FACT-L	Lower HL: poorer general (p = 0.001) and disease related QOL (p<0.001, significant also in regression analysis), no significant association with self-care behaviours (p = 0.093)	Higher (4)
Lillie, 2007, USA [34]	163 36–87 years 100%	Breast, stage I-II	Preference for participation in decision making Retention of information	Measures developed for study Adapted response scale from the Control Preferences Scale	Higher HL: greater number of correct answers (p<0.01), preference for more active participation in decision making (p = 0.03 in unadjusted analysis)	Higher (4)
Lim, 2019, Australia [35]	68 NR 62% (42)	Multiple, stage not reported	Cancer care coordination	Cancer Care Coordination Questionnaire (CCCQ)	Higher HL: better experience of cancer care coordination (p<0.001)	Higher (4)
Mahal, 2015, USA [36]	375 NR 0%	Prostate, stage not reported–biochemical recurrence	Unproven use of early salvage androgen deprivation therapy (ADT)	Three validated questions developed as a part of the Memorial Anxiety Scale for Prostate Cancer (MAX-PC) index.	Higher HL: less likely to undergo salvage ADT (p = 0.016, non-significant in multivariate analysis p = 0.07)	Higher (4)
Matsuyama, 2011, USA [37]	138 21–80 years 62% (86)	Multiple, stage II-IV	Information needs	Adapted Toronto Informational Needs Questionnaire (TINQ)	Lower HL: greater total (p<0.05), psychosocial and tangible information needs (both p<0.01 in bivariate analysis)	Higher (4)
McDougall, 2018, US [38]	277 NR 47% (130)	Colorectal, ‘localised or regional’	Cancer treatment related financial hardship Non-adherence to surveillance guidelines	Measures developed for study including questions from Medical Expenditure Panel Survey (MEPS) Experiences with Cancer Supplement	Lower HL: greater financial hardship (p<0.05), no association with adherence to surveillance colonoscopy	Higher (4)
McDougall, 2019, USA [39]	301 31–75 years 48% (143)	Colorectal, ‘localised or regional’	Health related quality of life	Specific PROMIS Short Forms	Lower HL: higher pain interference, higher sleep disturbance and higher depression scores (all p<0.05 in multivariate analysis)	Higher (4)
Mohan, 2009, USA [40]	184 NR 0%	Prostate, T1a-T2c	Perceived decrease in longevity with observation (PDLO) Perceived increase in longevity with treatment (PILT)	PDLO and PILT calculated from self-assessment of life expectancy and Charlston Comorbidity Index to estimate baseline comorbidity adjusted life expectancy	PDLO and PILT not associated with HL	Higher (4)
Ousseine, 2020, France [41]	4045 NR 63%	Multiple, stage not reported	Medico-social follow up Cancer related fatigue Depression and anxiety Sequelae following treatment	Questions developed for study Fatigue subscale of EORTC QLQ HADS	Lower HL: increased likelihood of follow up by GP and contact with social worker (in multivariable analysis), higher anxiety, depression, fatigue and sequelae following treatment (all p<0.001)	Higher (4)
Ozkaraman, 2019, Turkey [42]	111 NR 75% (83)	Multiple, stage I-IV	Quality of life Self-efficacy	Self-Efficacy to Manage Chronic Disease (SEMCD) scale EORTC QLQ-C30	Lower HL: poorer general QOL (p = 0.036) and increased symptom subscale score (p = <0.001), no significant association with self-efficacy	Higher (4)
Plummer, 2017, Australia [43]	36 39–69 years 100%	Breast, stage I-IV	Physical activity	Questions from Active Australia Survey.	Higher HL: greater physical activity (p<0.01)	Higher (4)
Polite, 2019, USA [44]	120 NR 33% (39)	Lung, gastric and pancreatic, stage not reported	Clinical trial attitudes, knowledge, and interest Preference for decision making	24 items from previously developed clinical trial questionnaire Adapted Control Preferences Scale	Higher HL: increased willingness to take part in a clinical trial if offered (p = 0.049), no significant association with decision-making preferences	Higher (4)
Post, 2020, USA [45]	298 NR 99% (285)	Breast, stage 0-III	Patient engagement (knowing participation in change, patient activation)	Knowing Participation in Change Short Form (KPC-SF) 10-item Patient Activation Measure (PAM-10)	Higher HL: greater patient engagement (p≤0.001 in bivariate analysis only)	Higher (4)
Tagai, 2020, USA [46]	431 42–86 years 0%	Prostate, stage not reported	Self-efficacy for re-entry Perceptions of medical interactions Practical concerns	Measures developed for study incorporating 5-item scale from Cancer Rehabilitation Evaluation System	Higher HL: greater self-efficacy for re-entry (p<0.001) and fewer practical concerns (p<0.05 in multivariable analysis)	Higher (4)
Xia, 2019, China [47]	4589 NR 77% (3532)	Multiple, stage not reported	Quality of life	EORTC QLQ-C30	Lower HL: poorer QOL (p<0.001 in logistic regression analysis)	Higher (4)
Anderson, 2021, USA [48]	183 NR 100%	Multiple, stages I-III	Impact of cancer self-management on psychosocial functioning Perceived general health	Measures developed for study PETS PROMIS Global-10	Lower HL: higher psychosocial impact score (p<0.05) with indirect effect on general physical and mental health	Lower (3)
Bol, 2018, The Netherlands [49]	197 65–86 years 35% (69)	Multiple, all stages	Recall of information	Questions developed for study based on the Netherlands Patient Information Recall Questionnaire (NPIRQ).	Higher HL: higher recall (p = 0.016 in multiple linear regression analysis)	Lower (3)
Douma, 2012, The Netherlands [50]	104 28–86 years 40% (42)	Multiple, stage not reported	Information needs	Information Preferences of Radiotherapy Patients Questionnaire (IPRP)	Lower HL: greater decrease in need for information about treatment over time (p = 0.05)	Lower (3)
Gonderen Cakmak, 2020, Turkey [51]	100 NR 57% (57)	Multiple, stage not reported	Oral chemotherapy adherence	Oral Chemotherapy Adherence Scale (OCAS)	Higher HL: higher medication adherence (p = 0.000)	Lower (3)
Goodwin, 2018, Australia [52]	565 NR 0%	Prostate, stage not reported	Quality of life	SF-36	Higher HL: better mental health status (p<0.01), weaker associations with physical health status (p<0.01)	Lower (3)
Gunn, 2020, USA [53]	228 NR 100%	Breast, all stages	Cancer related needs Patient self-efficacy	Adapted Cancer Needs Distress Inventory (CaNDI) instrument Communication and Attitudinal Self-Efficacy scale for cancer (CASE-cancer)	Lower HL: higher cancer-related needs at baseline (p<0.05 in multivariable analysis), lower self-efficacy at baseline (p<0.05)	Lower (3)
Gupta, 2020, India [54]	224 NR 55% (123)	Multiple, stage not reported	Adverse drug reactions	Identified by study investigator, graded and causality established	Lower HL: higher grade 3 and above adverse drug reactions (p<0.0001 in bivariate analysis)	Lower (3)
Halbach, 2016, Germany [55]	413 65–88 years 100%	Breast, stage 0-IV	Fear of progression	FoP-Q-SF	Lower HL: higher FoP (p<0.05)	Lower (3)
Heß, 2020, Germany [56]	449 23–89 years 63% (284)	Breast, prostate, colorectal, stage not reported	Unexpressed needs	Measures developed for study	Lower HL: higher unexpressed needs (p<0.05)	Lower (3)
Heuser, 2019, Germany [57]	863 NR 100%	Breast, stage 0-IV	Participation in multidisciplinary tumour conferences	Patient self-report of offer to participate and acceptance of this offer	Lower HL: less likely to participate in MTCs (p<0.05)	Lower (3)
Joyce, 2020, USA [58]	38 NR 0%	Prostate, stage not reported	Treatment regret	Measured using previously developed items	Lower HL: higher treatment regret (p<0.05).	Lower (3)
Kappa, 2017, USA [59]	504 NR 16%	Bladder, stage not reported	Use of post-operative discharge services	Medical records	Lower HL: greater use of discharge services (p = 0.016, non-significant in multivariable analysis)	Lower (3)
Kim, 2001, USA [60]	30 NR 0%	Prostate, all stages	Prostate cancer knowledge	Measures developed for study	Higher HL: higher prostate cancer knowledge (p = 0.0001, bivariate analysis)	Lower (3)
Nakata, 2020, Germany [61]	927 NR 100%	Breast, stage 0-IV	Need for psycho-oncological care	FoP-Q-SF Adapted subscale of the WIN-ON-Questionnaire	Lower HL: more likely to develop a need for psychological support (p = 0.003 in multiple regression analysis)	Lower (3)
Parker, 2020, USA [62]	46 NR 100%	Breast, stage I-III	Chemotherapy knowledge	Leuven Questionnaire on Patient Knowledge of Chemotherapy (L-PaKC)	Higher HL: greater chemotherapy knowledge (p<0.05 in univariate analysis)	Lower (3)
Scarpato, 2016, USA [63]	368 NR NR	Bladder, pT0-4	Post-operative complications Readmission	Medical records review	Lower HL: increased risk of developing minor complication (p<0.05 in multivariable regression analysis), no significant association with time to first ED visit or readmission	Lower (3)
Smith, 2020, Australia [64]	150 NR 71% (106)	Multiple, stage not reported	Knowledge and attitudes regarding clinical trials	Knowledge and Attitudinal Barrier Survey	Higher HL: better trials knowledge (p = 0.04 in multivariable regression analysis)	Lower (3)
Song, 2012, USA [65]	1581 41–79 years 0%	Prostate, T1-T2	Health related quality of life	SF-12	Lower HL: lower physical wellbeing (p<0.0001, non-significant in multivariable analysis) and poorer mental wellbeing (p = 0.0394 in adjusted model)	Lower (3)
Watson, 2020, USA [66]	100 NR 100%	Gynae-cological, not reported	Medication adherence	Validated three item measure	Adherence not significantly associated with HL	Lower (3)
Yen, 2020, USA [67]	311 NR 100%	Breast, stage not reported	Observed shared decision making	OPTION-5	Observed shared decision-making not significantly associated with HL	Lower (3)
Eton, 2019, USA [68]	91 31–92 years 59% (54)	Multiple, stage not reported	Health related quality of life Treatment burden	Global physical and mental health summary scores of PROMIS-10 Role-social activity limitations and physical/mental exhaustion scales of PETS	Lower HL: greater physical/mental exhaustion (p = 0.01 in linear regression analysis), and lower 6-month physical wellbeing (<0.05 in bivariate analysis)	Lower (2)
Halbach, 2016, Germany [69]	1060 21–88 years 97% (1023)	Breast, stage 0-IV	Unmet information needs	Modified version of Cancer Patients Information Needs (CaPIN)	Lower HL: higher unmet information needs (p<0.01)	Lower (2)
Janz, 2017, USA [70]	1295 NR 100%	Breast, stage I-II	Doctor-patient communication regarding risk	Questions developed for study	Patient perception of whether doctor discussed recurrence risk not significantly associated with HL	Lower (2)
Rust, 2015, USA [71]	48 NR 100%	Breast, stage not reported	Medication self-efficacy and adherence	Adherence to Refills and Medications Scale (ARMS) Self-Efficacy for Appropriate Medication Use Scale (SEAMS)	Higher HL: higher medication adherence and self-efficacy for medication use (p = 0.044 and p = 0.027 in linear regression analysis)	Lower (2)
Turkoglu, 2019, Turkey [72]	126 35–89 years 12% (15)	Bladder, non-muscle invasive	Compliance with cystoscopic follow up and treatment as per protocol	Unclear	Higher HL: higher treatment continuity rate (p = 0.008 in bivariate analysis)	Lower (2)
Wolpin, 2016, USA [73]	26 NR 0%	Prostate, localised	Eye tracking patterns	Usability measured with Tobii T60 eye tracker and an observer form	Lower HL: more time spent on prognostic text and infographic	Lower (2)

* Includes adults with cancer only. Multiple refers to more than three tumour sites. Abbreviations: EORTC QLQ-C30: European Organisation for Research and Treatment of Cancer Quality of Life Questionnaire C30; FACT: Functional Assessment of Cancer Therapy (-B: Breast, -G: General, -HN: Head and neck; -L: Lung); FoP-Q-SF: Short version of the Fear of Progression Questionnaire; HADS: Hospital Anxiety and Depression Scale; HL: health literacy; HRQOL: Health related quality of life; NR: not reported; PETS: Patient Experience with Treatment and Self-Management framework; PROMIS-10: Patient-Reported Outcomes Measurement Information System-10; QOL: Quality of life; SF-12/36: 12/36-item Short Form Health Survey.

**Table 2:** Association of outcomes to health literacy reported by included studies.

Category	Association	Outcomes
Information processing	Lower health literacy:	Lower understanding [16, 25]
		Poorer estimation of recurrence risk [16]
		Greater information needs and greater decrease in needs over time [37, 50, 69]
		More time spent on prognostic information and infographic (eye tracking) [73]
	Higher health literacy:	Higher recall [34, 49]
		Greater knowledge [20, 60, 62, 64]
Decision making	Higher health literacy:	Preference for more active participation [34]
		Higher perceived involvement [28]
	No association:	Preference for more active participation [44]
		Observed shared decision making [67]
Quality of life	Lower health literacy:	Poorer quality of life [19, 23, 29, 30, 33, 39, 42, 47, 48, 52, 65, 68]
	No association:	Quality of life [18]
Treatment and health service use	Lower health literacy:	Increased number and length of hospital admissions [17]
		Increased likelihood of GP follow up for cancer [41]
		Increased use of post-operative discharge services [59]
		Increased likelihood of treatment complications [54, 63]
	Higher health literacy:	Increased odds of receiving chemotherapy [26]
		Increased likelihood of breast reconstruction [24]
		Lower likelihood of receiving unproven treatment [36]
		Greater treatment continuity [72]
	No association:	Hospital admissions and emergency department visits [63]
		Adherence to recommended follow up [38]
Medication adherence	Higher health literacy:	Higher medication adherence [21, 51, 71]
	No association:	Medication adherence [66]
Care coordination	Lower health literacy:	Poorer experience of care coordination [35]
		Greater requirement for patient navigation assistance [31]
		Lower likelihood of patient participation in multidisciplinary tumour conferences [57]
Other	Lower health literacy:	Lower levels of physical activity [19, 43]
		Higher cancer related and unexpressed needs [53, 56]
		Greater need for psychological support [61]
		Increased financial hardship [38]
		Increased fear of progression or recurrence [30, 55]
		Greater treatment regret [58]
		Lower self-care management [32]
		Greater distress [19, 22, 41]
		Increased upper extremity disability after breast cancer [29]
	Higher health literacy:	Greater self-efficacy [46, 53]
		Preference for patient centred care [27]
		Greater patient engagement [45]
		Fewer practical concerns [46]
		Increased willingness to participate in a clinical trial if offered [44]
	No association:	Self-efficacy [42]
		Mortality [26]
		Distress [22]
		Perception of doctors’ communication of recurrence risk [70]
		Perceived changes to longevity with treatment or observation [40]
		Presentation with early stage disease [26]
		Self-care behaviours [33]

#### Information processing

Five higher and seven lower quality studies considered outcomes relating to information processing. Lower health literacy was associated with lower ease of understanding, as well as higher and more variable estimates of risk relating to breast cancer recurrence in women with early stage disease [16, 25]. Participants with lower health literacy had significantly higher unmet information needs in another large (*N =* 1060) study of patients with newly diagnosed breast cancer, although confounding variables were not controlled for [69]. A smaller but higher quality study of patients with mixed tumour sites, which did consider confounders, reported a significant association between health literacy and information needs in bivariate analysis only [37]. For radiotherapy outpatients with lower health literacy, the need for information about treatment at a single centre decreased significantly from pre-initial consultation to 3–5 weeks after the initial visit [50], though again, confounders, including time between consultations and treatment course length, were not accounted for.

Higher health literacy was associated with higher information recall in patients with breast cancer and in older patients with mixed tumour sites [34, 49]. It was also associated with greater disease specific knowledge about human papilloma virus (HPV) among patients with head and neck cancer [20], greater prostate cancer knowledge in patients with the disease [60], trials knowledge [64] and, in a small single centre study, with chemotherapy knowledge [62]. A small study of patterns of eye tracking reported a difference between time spent on aspects of a web based prostate cancer decision aid by those with higher and lower health literacy [73]. Those with lower health literacy appeared to spend longer on the prognostic text and infographic, but this was based on very limited data from 12 participants.

#### Decision making

Four studies exploring health literacy and decision making (three of higher quality) found mixed results. Using self-report measures, an association between higher health literacy and preference for more active participation in decision making was reported in one study of women making decisions about breast cancer recurrence risk testing [34], and with higher perceived involvement in shared decision making in another cross-sectional study of cancer patients at a single centre [28]. Yet no association was found when assessing preference for involvement in decision making regarding participation in clinical trials [44]. A secondary analysis of data from a randomised controlled trial evaluating decision aids for breast cancer, the only study to measure observed shared decision making, did not find a difference according to health literacy [67]. The chosen cut point for the health literacy screening question was higher than is recommended [74, 75], with a higher sensitivity but lower specificity for detecting lower health literacy, which may account for the lack of difference seen.

#### Quality of life

Twelve studies, of which eight were higher quality and five had over 500 participants, reported an association between lower health literacy and poorer quality of life. Studies included patients with colorectal [19, 39], breast [29], prostate [52, 65], lung [33], head and neck [23, 30] and mixed tumour sites [42, 47, 48, 68], and used a variety of health literacy and quality of life assessment tools. Only a single survey did not find a significant difference in quality of life between patients with low and higher health literacy [18], which may be due to its relatively small sample size compared with other large higher quality studies [19, 29, 47]. The study included a convenience sample of 97 patients with mixed tumour sites recruited from the waiting rooms of two clinics, and assessed health literacy using three different tools. Lower health literacy ranged from 5%-46% using the different measures, though the authors note there was no association between any measure and quality of life.

#### Treatment and health service use

Eleven studies considered treatment and health service use, six of which were higher quality. Higher health literacy was significantly associated with increased odds of receiving adjuvant chemotherapy for stage III/IV colorectal cancer [26]. It was also associated with an increased likelihood of reconstruction after mastectomy in a cross sectional study of 336 women with breast cancer attending a single centre [24], though this was significant in univariate analysis only. A further study of men with prostate cancer identified a trend for those with higher health literacy having a lower likelihood of undergoing unproven salvage androgen deprivation therapy for prostate specific antigen (PSA) recurrence [36], but this was again significant in univariable analysis only. In a single centre study of patients receiving chemotherapy, those with lower health literacy experienced more grade 3 and above adverse drug reactions [54].

Although no association was found in one retrospective study [63], lower health literacy was significantly associated with increased number and length of hospital admissions in a cohort study of patients with mixed tumour sites (*N =* 752) [17] after controlling for diagnosis, receipt of chemotherapy, comorbidities and other variables. In a national survey of 4045 French cancer survivors 5 years post diagnosis, those with lower health literacy were more likely to see their general practitioner for follow up of their cancer, which may suggest increased health service use, though data on frequency, reasons for visits, and contact with a specialist was not collected [41].

Patients requiring post-cystectomy discharge services in one centre had lower health literacy scores; significant on bivariate analysis [59], however, a change in practice during the study period led to an increase in the number of patients receiving discharge services regardless of risk factors is likely to have affected outcomes. In the same centre, those with lower health literacy were significantly more likely to experience a minor post-operative complication [63]. Treatment continuity for patients with non-muscle invasive bladder cancer was significantly higher in those with adequate health literacy in another study [72], but it is not clear how this was assessed, and confounders were not controlled for in the analysis. In another study, self-reported adherence to follow up after bowel cancer was not associated with health literacy [38].

#### Medication adherence

Four studies, of which one was higher quality [21], explored the association between health literacy and oral medication adherence. Adherence to general medications [71], specific oral chemotherapy (capecitabine) [21], and to various anti-cancer medications, including hormonal and targeted treatments [51, 66] was assessed. Higher health literacy was associated with higher levels of adherence in three studies of up to 100 participants [21, 51, 71]. One study of patients with gynaecological cancers (*N =* 100) did not report a significant association, though it was not powered to detect predictors of non-adherence [66]. All but one study [21] relied on self-report.

#### Care coordination

Three studies considered aspects of care coordination, of which two were small but considered higher quality [31, 35]. One survey of Chinese migrants with cancer in Australia found a positive correlation between higher health literacy and better experience of care coordination [35]. Another, of patients with mixed tumour sites [31], found an association between lower health literacy and higher input required from a patient navigator, although this did not remain significant in multivariate analysis. The third study involving 863 women with breast cancer found that those with ‘inadequate’ health literacy, as determined by the HLS-EU-Q16, were significantly less likely to participate in multidisciplinary tumour conferences than those with ‘sufficient’ health literacy [57].

#### Other outcomes

A range of other outcomes were also explored. Lower health literacy was associated with lower levels of physical activity, significant on bivariate analysis in a large study of patients with colorectal cancer [19], and in stepwise regression analysis of patients with breast cancer [43], and with significantly increased upper extremity disability after breast cancer in bivariable analysis [29]. It was also associated with higher cancer-related [53] and unexpressed needs [56], increased likelihood of need for psychological support [61], increased fear of progression in a study of older patients with breast cancer [55], higher fear of recurrence in patients with head and neck cancers [30], and greater treatment regret in a small study of men with prostate cancer [58]. Lower health literacy was associated with greater distress in three studies [19, 22, 41], though the same association was not found when one of the studies used a different measure of health literacy [22]. Lower health literacy was significantly and independently associated with increased cancer treatment related financial hardship [38], and self-care management scores were lower for patients with lower health literacy in another single centre study [32]. Higher health literacy was associated with greater preference for patient centred care [27], patient engagement [45], and self-efficacy in two studies [46, 53], though no association was found in a third single-centre study [42]. Those with higher health literacy were significantly more likely to report willingness to participate in a clinical trial if one was offered [44], and men with early prostate cancer and higher health literacy reported significantly fewer practical concerns [46].

Mortality [26], presentation with early stage disease [26], self-care behaviours [33], perception of doctors’ communication of recurrence risk [70] and perceived changes to longevity with treatment or observation [40] were not associated with health literacy.

### Objective 2: Qualitative studies exploring the role and consequences of health literacy as patients with cancer access, understand, appraise and use information and services to make decisions about health

Eight qualitative studies were identified and add the patient voice to the findings of this review (Table 3). Studies included patients with prostate cancer [76–78], breast cancer [79, 80] and haematological malignancies [81]. One included patients with different primary tumours [82] and one study did not report on tumour site [83]. Six studies were of higher quality according to the MMAT, and one mixed methods study scored highly for the qualitative component but achieved a lower score overall.

**Table 3:** Qualitative studies exploring the role of health literacy in patients to access, understand, appraise and use information and services to make decisions about health.

First author, year, location	Aim/objectives	Study design	Sample characteristics (number, tumour sites, age range, sex)	Key themes and findings	MMAT score
Burks, 2020, USA [80]	To assess the perceptions of risks, benefits, and the informed consent process for patients already enrolled in a phase 2 clinical trial using intraoperative radiation therapy (IORT) with a nested study exploring how the perceptions of risks and benefits of clinical trial enrolment differed based on varying levels of health literacy	Structured interviews with convenience sample of participants already recruited to phase 2 parent study. Health literacy assessed using screening questions.	20 participants, early stage breast cancer, 45–90 years, 100% female	Weight of risks and benefits Pragmatic decision making Confidence in provider recommendation	5
Cohen, 2013, USA [81]	To describe the meaning of patients’ experiences with hematopoietic stem cell transplantation (HSCT), with a focus on health literacy.	Interviews using open ended questions conducted at five time points from pre-transplantation to 100 days post.	60 participants, haematological malignancies, undergoing stem cell transplant, 22–71 years, 50% female	They did not tell me Decision dilemmas Fears of dying Tough symptoms and side effects Relying on others	5
Kayser, 2015, Denmark [76]	To explore whether the scores of and responses to a Health Literacy Questionnaire (HLQ) can be used to identify individuals in need of information and support, to reveal differences in perception and understanding in health related situations within couples and to explore whether the health literacy domains constituting the HLQ emerged as themes important to the men and their spouses.	Mixed methods approach. Patients and spouses interviewed separately using HLQ as framework for questioning.	8 patient participants, early stage prostate cancer, 55–70 years, 100% male	Involvement of their spouses and people around them Their support from and interaction with healthcare professionals Their use of the Internet for information retrieval	3
Martinez-Donate, 2013, USA [82]	To identify the health literacy barriers and patient navigation needs of rural cancer patients in Wisconsin using the Chronic Care Model as a guiding and integrative framework.	Mixed methods approach. Face to face semi structured interviews with patients from five centres. Health literacy assessment performed. Closed ended question survey later completed by telephone. Focus groups and surveys with clinical staff.	53 participants, multiple tumour sites (breast, lung, colorectal and prostate), 39–86 years, 63% female	Community Characteristics Self-management support Delivery System Design Decision Support	2
Oliffe, 2011, Canada [77]	To describe how men who attend prostate cancer support groups (PCSGs) engage with health literacy and consumerism.	Part of larger ethnographic study. Participant observation at support group meetings and fundraising events as well as individual interviews.	54 participants, prostate cancer, 53–87 years, 100% male	Numbers and measures as the foundation of prostate cancer literacy Group information processing Shopping around	5
Rust, 2011, USA [79]	To explore the issues of health literacy and medication adherence among underserved breast cancer survivors	Two focus groups containing 12 participants each.	24 participants, breast cancer, age range not reported, 100% female	Inequality of access to health information Acquisition of medication information Medication usage and adherence Barriers to access to medications	5
Treloar, 2013, Australia [83]	To understand and integrate the perspectives of Aboriginal people, their carers and health workers regarding the health literacy required for engaging with cancer screening, diagnosis, care and treatment.	Semi-structured in-depth interviews with patients, carers and healthcare workers	22 patient participants, tumour sites and age range not reported, 73% female	Recognising susceptibility to cancer Recognising opportunities to learn from each other Opportunities for practical services and programmes for health literacy in relation to cancer	5
Zanchetta, 2007, Canada [78]	To describe, analyse, and understand the participants’ ways of understanding and dealing with PC-related information as demonstrated by their informational strategies.	Open-ended, semi-structured interviews, participants’ personal journals, personal documents, genograms and ecomaps, interviewer’s observational notes, and observation of nonverbal cues during the interviews.	15 participants, localised prostate cancer, 61–83 years, 100% male	Social and informational networks Overcoming professional medical language Spiritual and emotional influences Literacy levels Silence among men Deductive and hypothetical reasoning	4

Table 4 demonstrates how the original themes reported by the individual studies were grouped to form meta-themes. The meta-themes identified included situational influences (networks and system), personal influences, information processing, and consequences of health literacy. Situational influences refer to the factors external to the person which influence their ability to process information. They include network influences, incorporating sources of information and support outside of the healthcare environment, as well as system influences, relating to professionals within the healthcare system and structural factors involved in care delivery. Personal influences refer to more internal factors that might contribute to health literacy, such as prior experience, cultural values and emotions. Information processing encompasses the strategies described by patients to help them deal with and process the information they face. Consequences refer to the outcomes of these influences and processing, and include negative aspects, such as fear or uncertainty, as well as more positive outcomes, such as empowerment and better understanding.

**Table 4:** Meta-themes and the contributory themes extracted from original papers.

Meta-themes	Themes from original papers
Situational influences	Relying on others [81]
• Networks	Involvement of their spouses and the people around them [76]
	Group information processing [77]
	Recognising opportunities to learn from each other [83]
	Social and informational networks [78]
	Their use of the internet for information retrieval [76]
	Pragmatic decision making [80]
Situational influences	Overcoming professional medical language [78]
• System	Self-management support [82]
	Delivery system design [82]
	Support from and interaction with healthcare professionals [76]
	Opportunities for practical services and programmes for health literacy in relation to cancer [83]
	Inequality of access to health information [79]
	They did not tell me [81]
	Decision support [82]
	Confidence in provider recommendation [80]
Personal influences	Recognising susceptibility to cancer [83]
	Community characteristics [82]
	Spiritual and emotional influences [78]
	Literacy levels [78]
	Silence among men [78]
Information processing	Numbers and measures as the foundation of prostate cancer literacy [77]
	Deductive and hypothetical reasoning [78]
	Weight of risks and benefits [80]
Consequences	Shopping around [77]
	Decision dilemmas [81]
	Fears of dying [81]
	Tough symptoms and side-effects [81]
	Medication usage and adherence [79]
	Acquisition of medication information [79]

#### Situational influences

All eight papers described themes relevant to the role of external or situational influences on health literacy. Two key areas were identified: the importance of networks, which were largely supportive and facilitated understanding; and the system, which often acted as a barrier and inhibitor to the development of health literacy.

#### Situational influences—Networks

Social and informational networks played important roles as facilitators of health literacy and were among the situational resources available to patients enabling them to access, understand, appraise and use information and services. Although some participants expressed a preference to deal with their diagnosis by themselves [76], many relied on friends and family as sources of information and support [76–78, 80, 81]: *“I was a little reluctant because I really didn’t know that much about the IORT at first*. *But then I talked*, *actually after talking to a friend of mine who had*, *you know*, *the traditional radiation*, *she said*, *“Man*, *I can’t imagine how much better it would be just to do it once*, *just to have one dose of radiation*.*”… So after talking to my friend who had a very bad experience*, *she got burned… I just decided I didn’t want to do the traditional”* (female phase 2 clinical trial participant, adequate health literacy) [80]. Learning from other patients about their experiences, often through support groups or organisations, allowed participants to develop a greater understanding of their diagnosis and treatment [77, 78, 81]. Support groups also offered participants the opportunity to hear from and talk to ‘experts’ outside the consultation setting, helping to build confidence to ask questions: *“You find confidence and get encouraged to talk to health professionals*, *ask questions*, *and that will only come through building confidence*. *If you have any problem*, *try to seek the answer for it”* (73 year old attendee at prostate cancer support group for 14 years) [77]. Only occasionally, these social connections acted as barriers, such as when the knowledge imparted was inaccurate or led to increased fear [83]. The internet was a valuable resource for many participants, who were able to use it to find further information and additional support [76, 77, 80, 81]: *“I have done research through the Internet*. *The Leukemia Society*, *I called them*, *and they got me*, *they hooked me up with another patient that had gone through all of this*, *and she and I talked back and forth on the phone*. *She told me about talking to other patients at the hospital*. *I am a member of a support group on the Internet that we counsel leukemia and everything*, *and every kind of research that you can think of*, *I have read about it*. *So*, *when the doctors come in and talk to me*, *it is nothing unknown or shocking to me because I have read about it*” (41-year-old African American woman prior to admission for stem cell transplant) [81].

#### Situational influences—System

Health literacy was also influenced by ‘system’ factors that are outside participants’ control. Professionals within the healthcare system played a vital role in imparting information, and, when done well, participants’ confidence in their clinicians made them feel more comfortable in their decision making [80]: *“I was concerned and I was very open*, *and they were open with me in explaining what the procedure would be… I almost made it right there on the spot because I just felt so secure that my problem would be taken care of”* (female phase 2 clinical trial participant, marginal health literacy) [80]. Yet this information giving was not always done in a way that participants could understand [78, 82]: *“They used too many big words… It is a complicated procedure*. *They explained everything*, *but you still don’t get it*.*”* (35 year old postal carrier undergoing stem cell transplant) [81]. The healthcare system itself placed high demands on participants’ health literacy, with over-complicated forms which some participants signed without fully understanding: *“I have signed a lot of papers without reading*. *I figure they ain’t gonna give me nothing to sign if it’s bad”* (rural cancer patient) [82], and through inconsistent access to resources and opportunities to further understanding [79]. Participants in one study described cancer care as a “foreign” experience and didn’t know what to ask about their treatment options [82], providing support more generally for the recommendations by Treloar et al. [83] for improved community education to raise awareness and help prepare people for such a diagnosis.

#### Personal influences

Participants’ health literacy was also affected by personal influences. These included cultural and community values such as stoicism, which led to patients ‘suffering in silence’ rather than ‘bothering’ healthcare professionals [82] and a “silence among men” impeding open discussion and thus understanding [78]. Silence was exacerbated by limited experience of cancer prior to diagnosis: *“Cancer has never sort of crossed my life till now… I used to be a health worker*, *an educator*, *but cancer was never part of my life*, *I never knew anyone with cancer*, *I never seen anyone with cancer*, *maybe on TV but not in the here and now*, *cos I was always busy with Aboriginal health and teaching Aboriginal health*, *but cancer was never part of our programme*, *which was a shame”* (Aboriginal patient who had previously worked in health sector) [83]. Participants’ general literacy was influenced by social and cultural exposures over time, typically encouragement at school or at home, and fed into their approach to learning about their condition [78].

#### Information processing

Some participants used strategies to help them process information, highlighted by two studies of male attendees at prostate cancer support groups. Focussing on numbers relating to pathological grading or biomarkers and the relationship of these to treatment options facilitated understanding of prostate cancer and allowed men to assess their options: *“Researcher*: *In what ways did it [the prostate cancer support group] help you steer your treatment*? *Participant*: *By giving me information about how each of the approaches is and how it works*, *the long-term and short-term effects of each*, *the certainties and uncertainties around each one*, *and certainly the cure rate”* (59 year old attendee at prostate cancer support group for three years) [77]. In another study, patients used a process of deductive and hypothetical reasoning, comparing information from different sources, or comparing themselves with others, to further their understanding, monitor their response to treatment, and verify information given to them by healthcare professionals [78]. A different study, assessing perceptions of the risks and benefits of participation in a trial of a novel radiotherapy technique for breast cancer, found that many participants did not believe there were any risks, and most focussed instead on the positives, such as convenience of the treatment, which were influential in their decision to take part [80].

#### Consequences

The result of some of these influences and processes can be seen in the wider consequences of health literacy. Where there was conflicting advice or poor understanding, this led to decision dilemmas, and participants were prepared to accept a treatment without full comprehension as a way of moving on and progressing their care [81]. When the information patients needed was not given, or not in a way they could understand, they experienced greater fear: *“Many of the things you fear are those you don’t understand”* (42 year old industrial worker undergoing haematopoietic stem cell transplant) [81]. Poorer prior understanding also led to more unanticipated side effects [81], which in turn influenced decisions about medication adherence: *FGA*: *“I don’t take everything they give me*.*” “If it has too many side effects*, *I don’t take it*.*” FGB*: *“I didn’t take anything because I was afraid of the side effects”*. *FGB*: *“They tell you some of the side effects but they don’t tell you all the side effects”* (quotes from female African American participants with breast cancer from two focus groups (FGA and FGB)) [79].

Conversely, those who had developed a good understanding and the confidence to do so were able to effectively navigate the healthcare system and exercise their rights to ensure that they received ‘good care’: *“I felt this urologist was pushing me for surgery and I thought geez*, *I don’t have enough knowledge*, *I want data*. *So*, *I kept pushing him*, *to tell me where the groups [PCSGs] were and he was reluctant to tell me*, *but finally he agreed*, *and I went*, *and I never went back to this guy and I started my search and the prostate cancer groups were very instrumental in helping me to make my decision*. *They gave me knowledge*.*”* (63 year old attendee at prostate cancer support groups for 10 years) [77]. Patients accessed information in different ways, influenced by personal factors including the desire not to be a bother, and situational influences, such as time pressures on healthcare staff. When these influences were removed, if patients knew their pharmacists well or they appeared to have time to talk, for example, or if patients had the confidence to overcome these influences, it was possible for them to seek and obtain the information they needed [79].

### Combined synthesis and conceptual map

**Fig 2:**
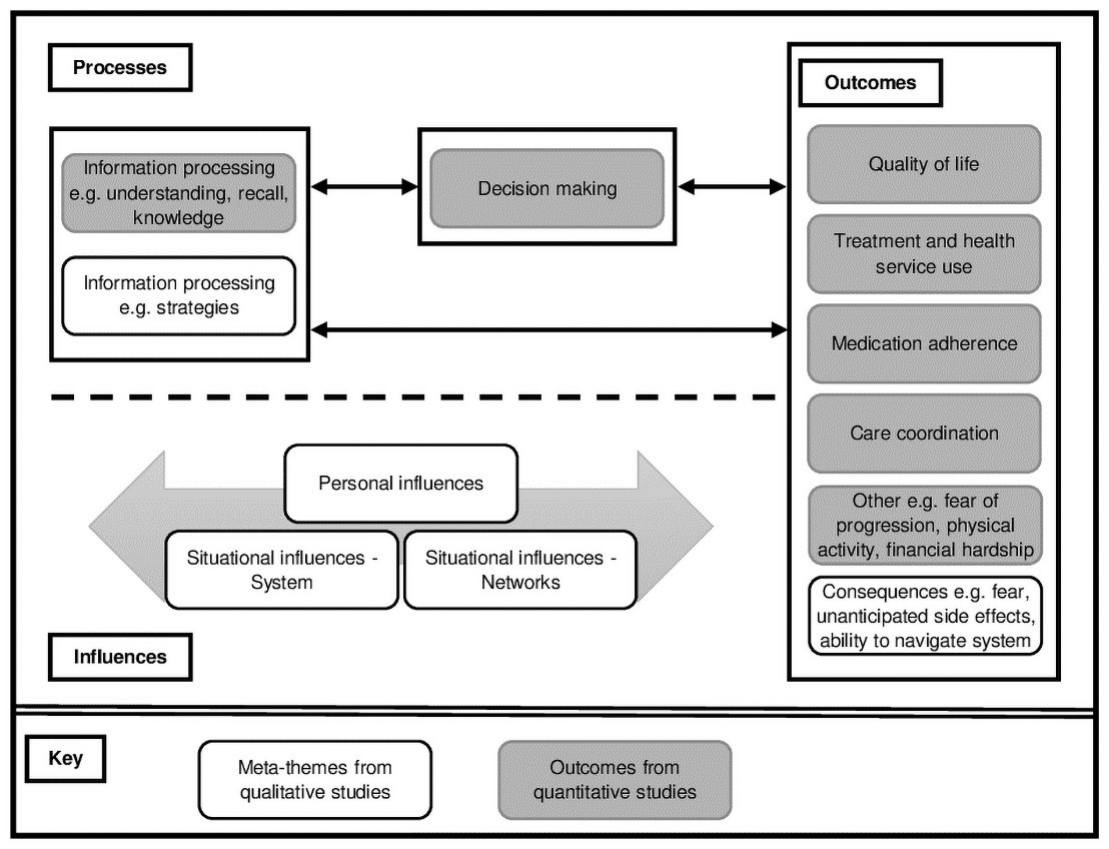
Concept map demonstrating links between findings from quantitative and qualitative data as ‘Processes’, ‘Outcomes’ and ‘Influences’ of health literacy.

The concept map below (Fig 2), shows the relationships between the outcomes associated with health literacy as identified by the quantitative studies (Objective 1) and the meta-themes identified from the qualitative synthesis (Objective 2). It draws on the existing framework developed by Edwards et al. [8] and the definition of health literacy proposed by the International Union for Health Promotion and Education [1].

Patients with lower health literacy may have more difficulty understanding and recalling the information they have been given, demonstrate lower knowledge and have higher unmet information needs (‘Processes’, Fig 2). The qualitative data suggest that situational influences, including the way that information is delivered, the complexity of the system, and the lack of resources available to patients make these tasks more challenging (‘Influences’). As such, patients are prepared to consent to treatments without fully understanding what they may entail, or what the potential risks and benefits are (‘Outcomes’). Fear and unanticipated side effects may arise as a consequence of lower health literacy through lack of understanding (‘Outcomes’) yet fear itself may influence and hinder comprehension (‘Influences’).

Those with higher health literacy are better able to process information (‘Processes’), engage more with health promoting activities such as exercise, and experience a better quality of life (‘Outcomes’). They may be more likely to seek out the additional information they need, perhaps learning to interpret numbers relating to their condition or finding opportunities to learn from others (‘Processes’). With greater understanding and knowledge of their disease and their rights, they may take a more active role in making decisions about their care and have greater confidence in navigating the system (‘Outcomes’).

## Discussion

The findings from this mixed studies systematic review demonstrate the role and consequences of health literacy in the oncology setting. The outcomes associated with health literacy are varied, with some having clear implications for care delivery, and others demonstrating the negative impact of health literacy difficulties on the experience of care as reported by patients themselves. While the quantitative data gives evidence for the measurable outcomes associated with health literacy, the qualitative findings complement this by adding the patient voice, identifying some of the influences of health literacy, and offering an insight into some of the associations seen. Findings relating to information processing and decision-making highlight some of the ‘Processes’ affected by these ‘Influences’ and demonstrate how health literacy may link to the described ‘Outcomes’. Ensuring that the system is considerate of the burden it places on patients, taking steps to simplify information and processes, providing patients with the confidence and opportunities to speak up, and making support available is therefore essential.

Although further empirical work is needed to determine the nature of these associations, the causal links between health literacy and health outcomes have been hypothesised [84, 85]. These models consider the range of mediating factors that may influence the pathway, including patient and system factors affecting access and utilisation of health care, provider-patient interaction and self-care [84], as well as health status, attitudes, emotions, motivation, self-efficacy and ecological resources [85], some of which are included as associated outcomes in this review. In addition, health behaviours and outcomes may in turn influence these mediators and health literacy skills [85]. Poorer quality of life, for example, which was consistently associated with lower health literacy, may be linked with other outcomes identified in this review, such as increased fear, greater financial hardship or a worse experience of treatment, as such outcomes may influence or indeed act as mediators in the pathway. Whether improving health literacy itself leads to better quality of life is as yet unknown [86], and this is an important outcome for further study.

Our review supports the Health Literacy Pathway Model presented by Edwards et al. [8], which draws on Nutbeam’s conceptualisation of health literacy as an asset that can be developed over time [2]. The model incorporates internal and external influences that may positively or negatively affect a person’s health literacy [8], factors we have also found to be important in patients with a cancer diagnosis. Such patients face many new challenges at a highly emotional time. It is therefore crucial that the systems and networks are in place to support patients, making it easier for them to access, understand, appraise and use the information they want and need by removing as many additional barriers as possible. In doing this, patients are afforded the best chance of being able to develop and use their health literacy to take an active role in their health and make informed decisions based on what is important to them.

The decision-making preferences and degree to which patients with lower health literacy feel able to take on a more active role in the oncology setting require further study. But whether a patient wishes to be actively involved in decision making or prefers to be guided by their clinician, an understanding of the aims and potential risks of treatment are key to informed consent [87]. The General Medical Council (GMC) guidance for doctors in the United Kingdom highlights the importance of taking steps to facilitate understanding, acknowledging that patients have different information needs and may prefer to receive information in different formats [87]. Our findings suggest that this is not always achieved.

One limitation of this review is the exclusion of studies using measures relating to health literacy but referring only to literacy. This was to ensure that *health* literacy remained the subject of interest, but other studies may have been missed. Secondly, to achieve consistency in a field with a range of measures, it was agreed that only those health literacy assessment tools used in their validated form would be included. Although this excluded some studies using non-validated adaptations, it was deemed important in order to be able to draw any comparisons between studies. As found elsewhere, the range of health literacy measures and identification of participants with lower health literacy makes such comparisons difficult. Over half of the included studies were conducted in the USA, with none carried out in the UK, which may limit the relevance of some results to other healthcare settings.

A major strength of the review is the use of the mixed methods approach, bringing together a more comprehensive picture of health literacy in the oncology setting, incorporating the patient voice and allowing us to better understand the experience from the patients’ perspective. The broad inclusion criteria allowed us to identify the association between health literacy and a wider range of outcomes than has previously been addressed [10, 11]. Additionally, involvement of a multidisciplinary team of experienced researchers and clinicians at all stages of the review ensured consistency and rigour throughout the process.

## Conclusion

Health literacy plays a key role in cancer care, with important implications for patient experience and outcomes. Those with lower health literacy face greater difficulties processing information, report poorer psychological outcomes and experience a poorer quality of life, whilst those with higher health literacy appear better informed and able to take on a more active role in managing their health. Future interventions aimed at supporting person centred care in this setting should therefore take account of health literacy and consider the factors influencing its development. Further research is required to better understand the decision making processes and preferences of those with lower health literacy receiving care for cancer.

## Supporting information

S1 Table Supplementary data on included papers reporting associations with health literacy. (DOCX)

S1 File Full search strategy. (DOCX)

S2 File Protocol. (DOCX)

S1 Checklist PRISMA checklist. (DOCX)
