# Supplementary material for: The role of health literacy in cancer care: A mixed studies systematic review
Source: PLoS One. 2021 Nov 12;16(11):e0259815. doi: 10.1371/journal.pone.0259815 (PMC8589210; doi:10.1371/journal.pone.0259815)
Supplement: S1 Table — (DOCX) [file pone.0259815.s001.docx]

**S1 Table. Supplementary data on included papers reporting associations with health literacy**

| **Author, year, location** | **Aim/objectives** | **Study description** | **Association of health literacy with outcomes** |
| --- | --- | --- | --- |
| Anderson, 2021, USA[49] | To better understand the combined challenges of self-management for cancer and chronic health conditions faced by cancer survivors. | Survey of female cancer survivors with at least one comorbidity from two cancer centres | Lower HL was associated with higher psychosocial impact score (p<0.05) and an indirect effect on general physical and mental health (mediated by self-management impact) |
| Bol, 2018, The Netherlands[50] | To present an overview of factors that, according to previous studies, may be predictive of (online) information recall in older adults and empirically test which of these factors relate to recall of online information among an older cancer patient population. | Cross sectional online survey of older (>65 years) cancer patients from two hospitals, an online panel and a research panel. | Higher HL was associated with higher recall in multiple linear regression analysis (β=0.18, p=0.016) |
| Brewer, 2009, USA[17] | To examine how health literacy informs the way breast cancer survivors understand risk, especially when communicated in different formats. | Cross sectional self-administered questionnaire assessing risk communication formats with women post-surgery in single centre | Women with lower HL gave higher and more variable estimates of recurrence risk compared to those with higher HL (mean 52% v 30% respectively, p=0.01) and expressed lower ease of understanding (p<0.001) |
| Brewer, 2012, USA[26] | To assess whether the standard genomic report provided to doctors is a good approach for communicating results to patients and comparing risk communication formats of varying complexity | Cross sectional randomised study testing risk communication formats with women eligible for genomic recurrence risk test in single centre | Lower HL was associated with lower perceived understanding of test results (p=0.01) |
| Busch, 2015, USA[27] | To evaluate the role of health literacy in decisions related to cancer treatment and to estimate the impact of health literacy on patient outcomes | Cohort study taking random sample (stratified according to self-reported years of education) from larger prospective, multi-site, observational study | Having adequate HL increased the odds of receiving chemotherapy compared to those with marginal/inadequate HL (stage III/IV disease) (OR, 3.29; 95% CI, 1.23–8.80).  Having adequate HL did not increase the odds of presenting with early-stage disease (all stages) HL level was not significantly associated with death in univariate or multivariate analysis. |
| Cartwright, 2017, USA[18] | To examine the ability of health literacy to predict hospitalisation of cancer patients. | Secondary analysis of data from cancer patients within five years of first cancer diagnosis, with valid medical record data, participating in cohort study | There was a significant direct relationship between HL and number of inpatient hospital admissions (β=-0.041, p=0.009) and total number of days spent hospitalised (β=-0.028, p=.023). Direct effects between HL and number of 30-day readmissions did not reach significance. |
| Chan, 2020, Malaysia[28] | To determine the extent to which cancer health literacy is associated with patient-centeredness, and secondarily, to identify other modifiable predictors in a Malaysian setting. | Cohort survey of patients with cancer attending single cancer centre | Higher HL was significantly associated with preference for patient centred care in multiple linear regression analysis (p=0.001) |
| Chang, 2019, Taiwan[29] | To evaluate the progress in achieving patients’ HL and the implementation of shared decision making | Cross sectional descriptive study of patients with cancer attending single centre | Higher HL was significantly correlated with higher extent to which participants felt involved in shared decision making (r=0.258, p=0.004) |
| Chrischilles, 2019, USA[30] | To describe the relationship of modern treatment characteristics with QOL in a contemporary sample of breast cancer patients and quantify the potential mediating effect of upper extremity disability on this relationship. Because a variety of patient factors were expected to directly affect both upper extremity morbidity and QOL and must be accounted for, a secondary objective was to describe these relationships. | Cross sectional questionnaire and linked cancer registry data from random sample of patients with breast cancer at eight centres | Women with lower HL reported significantly more disability in bivariable analysis (p=0.0062).  Those with lower HL reported significantly lower QOL after adjusting for disability (p=0.0063). |
| Clarke, 2021, Ireland[31] | To investigate for the first time in a population-based sample of head and neck cancer survivors the sociodemographic and clinical profile of health literacy and associations between health literacy and HRQOL, self-management behaviours and fear of recurrence. | National postal survey of patients within 5 years of diagnosis | Lower HL was significantly associated with lower self-management behaviours across several self-management domains, and with lower functional wellbeing (Coef: −1.49; 95% CI −2.76 to −0.22: p=0.0220) and head and neck specific HRQOL (Coef −4.95; 95% CI −9.87 to −0.02: p=0.046) in adjusted regression models. Fear of recurrence was significantly higher in those with inadequate HL in the adjusted model (Coef 0.98; 95% CI 0.04 to 1.92: p=0.040) |
| Douma, 2012, The Netherlands[51] | To gain an understanding of how patients’ information needs change from visit to visit in order to be able to better tailor information giving. | Longitudinal questionnaire study of radiotherapy outpatients at single centre | Lower HL was associated with a greater decrease in need for information about treatment over time from initial visit to follow up (p=0.05, exp b=1.75, 95%CI=1.01–3.05) |
| Eton, 2019, USA[69] | To identify risk factors for poor health-related quality of life in multi-morbid adult cancer survivors and explore whether perceived treatment and self-management burden mediate any of these relationships. | Survey and records review, part of larger prospective study, of cancer patients with at least one other chronic comorbid condition | Lower HL was associated with more physical/mental exhaustion in bivariate and linear regression analysis (b=−0.25 p=0.01), and lower 6-month physical wellbeing in bivariate analysis (<0.05) |
| Gonderen Cakmak, 2020, Turkey[52] | To examine the relationship between health literacy and self‑report medication adherence of Turkish cancer patients receiving oral chemotherapy. | Questionnaire study of medical oncology outpatients attending a single centre | There was a positive and strong correlation between HL and medication adherence (r=0.707, p=0.000) |
| Goodwin, 2018, Australia[53] | To test the Health Literacy Questionnaire (HLQ) in a sample of men with prostate cancer and examine the components of health literacy that are most strongly associated with mental and physical health‐related quality of life in men with prostate cancer. | Cross sectional questionnaire study of members of prostate cancer support groups | Several HL subscales were associated with mental health status in linear regression analysis (all b > 0.250, p<0.01), with weaker associations between several HL scales and physical health status (all  b<0.200, p<0.01) |
| Gunn, 2020, USA[54] | To examine the associations among language, HL, cancer related needs and self-efficacy among a sample of patients with newly diagnosed breast cancer | Secondary analysis of data collected from randomised controlled trial at single centre | Inadequate and marginal HL were associated with higher average cancer-related needs scores at baseline in multivariable analysis (p<0.05), but differences resolved by 6 months. Having inadequate HL was associated with lower self-efficacy at baseline (p<0.05) |
| Gupta, 2020, India[55] | To examine the association between HL and cognition level on the severity of adverse drug reactions | Questionnaire study of patients receiving chemotherapy at single centre | Inadequate HL was significantly associated with grade 3 and above adverse drug reactions (p<0.0001) in bivariate analysis |
| Hahn, 2010, USA[19] | To evaluate patient attitudes towards literacy screening, agreement between literacy tests, and associations between literacy, informed consent comprehension, and health-related quality of life. | Cross sectional survey, convenience sample of cancer patients in waiting rooms of two clinics providing care to uninsured populations | There was no significant difference in FACT-G scores between the two HL groups (p=0.244). |
| Halbach, 2016, Germany[56] | To investigate the distribution of health literacy levels over the course of breast cancer treatment in elderly women newly diagnosed with breast cancer, to investigate the distribution of fear of progression (FoP) levels over the course of breast cancer treatment in elderly women newly diagnosed with breast cancer, and to analyse the association of health literacy with FoP over the course of breast cancer treatment in elderly women newly diagnosed with breast cancer adjusting for relevant socio-demographic, clinical and psychosocial factors. | Part of prospective, longitudinal, multicentre cohort study, assessment during inpatient stay and subsequent postal survey of older (>65 years) patients | Inadequate and problematic HL were significantly associated with higher levels of FoP in regression analysis (6.50 points, p=0.000 and 3.02 points, p= 0.001 respectively) |
| Halbach, 2016, Germany[70] | To investigate the content and amount of unmet information needs in newly diagnosed breast cancer patients over the course of cancer treatment, considering phases of hospital stay/breast cancer surgery, follow-up treatment and post treatment and to analyse the association of health literacy – considering health literacy as a multidimensional concept – with four different domains of unmet information needs in newly diagnosed breast cancer patients over the course of cancer treatment | Part of prospective, longitudinal, multicentre cohort study, assessment during inpatient stay and subsequent postal survey | Unmet information needs were significantly higher across all domains for patients with inadequate or problematic HL (all p<0.01, >20 point difference in mean score for inadequate v sufficient HL) |
| Hendren, 2011, USA[32] | To describe the barriers to health care faced by a diverse group of newly diagnosed breast and colorectal cancer patients and to compare these barriers between non-Hispanic white and minority patients | Analysis of data prospectively collected for multi-site randomised study of patient navigation, used survey data from navigation arm | HL was associated with log navigation time in univariate analysis (p=0.02) but did not remain significant in multivariate analysis. |
| Heß, 2020, Germany[57] | To explore cancer patients' unexpressed needs in the admission interview. To identify the proportion of cancer patients with unexpressed needs in the admission interview of inpatient rehabilitation, to examine topics of unexpressed needs and reasons for not expressing them, to explore associations between not expressing needs and sociodemographic variables, clinical variables, health-related variables, and patients' evaluations of the admission interview, and to examine whether patients not expressing needs may be identified using sociodemographic, clinical, and health-related information typically available in the admission interview. | Questionnaire study of patients admitted for inpatient cancer rehabilitation at single centre | There was a statistically significant small to medium correlation between HL and not expressing needs (p value not given, p<0.05 taken as statistically significant) |
| Heuser, 2019, Germany[58] | To analyse the impact of individual HL, sociodemographic and disease-related characteristics as well as the impact of the variation between breast cancer centres on patient participation in multidisciplinary tumour conferences (MTCs). | Part of a prospective, longitudinal, multicentre cohort study, assessment during inpatient stay and subsequent postal survey | Those who participated in MTCs had significantly higher HL than those who did not. In multivariate analysis, patients with inadequate HL were less likely to participate in MTCs than patients with sufficient HL (OR = 0.31, 95% CI = 0.1–0.9, p<0.05) |
| Husson, 2015, The Netherlands[20] | To examine the prevalence of subjective functional HL among colorectal cancer survivors; to explore the relation between functional HL and health behaviours, HRQOL, and mental distress; and to explore whether or not functional HL and health behaviours are independently associated with HRQOL and mental distress as an indication for the potential mediating role of health behaviour in the relation between HL and HRQOL/mental distress. | Longitudinal population-based survey from registry of patients | Patients with low HL were less likely to meet guidelines for physical activity than those with medium or high HL (25% v 11% v 6%, p<0.01). In multiple linear regression analysis, low HL was negatively associated with all HRQOL subscales (p<0.01) and positively with mental distress (p<0.01). Observed differences between HL groups were of large clinical importance for cognitive functioning, medium clinical importance for global QOL and physical and social functioning, and of small clinical importance for role and emotional functioning. |
| İlhan, 2020, Turkey[33] | To determine the relationship between health literacy and illness self-care management in individuals with cancer. | Cross sectional questionnaire of outpatients treated at single chemotherapy unit | Self-care management scores were significantly lower for those with inadequate HL (p<0.01). HL was a significant predictor of chronic illness self-care management in multiple regression analysis (b = –0.158, p=0.03). |
| Inglehart, 2016, USA[21] | To assess the health literacy, Human papilloma virus (HPV) knowledge and information source utilisation among patients newly diagnosed with HPV-positive and HPV-negative oral squamous cell carcinoma in order to begin to address their information needs and to facilitate patient access to desired information sources. | Sub-study of prospective cohort questionnaire study of patients at single cancer centre | HPV-related knowledge correlated positively with HL (Spearman’s rho correlation coefficient 0.23, p < 0.01). Information seeking behaviour trended towards higher HL but was not significant (p=0.06). |
| Janz, 2017, USA[71] | To characterise patients’ perceptions of doctor–patient discussions about risk of recurrence in a large, diverse population-based sample of women with early-stage invasive breast cancer, to determine if the amount of discussion, approach used, and/or assessment of worry during the communication effort are associated with patient understanding of risk, and to determine whether doctors’ approaches to communicating risk and addressing worry vary by the patient’s personal factors. | Cross sectional survey and data from registry database of women with early-stage breast cancer | HL was not significantly associated with patient perception of whether the doctor discussed recurrence risk (OR 0.87, 95% CI 0.55, 1.38) |
| Jiang, 2019, USA[22] | To explore how patient self-reported severity of oral anticancer agent (OAA)-related side effects and perceived effectiveness of side effect self-management were associated with adherence to OAAs, using capecitabine as the example. Relationships of adherence with other potential factors, such as patient socio-demographics, clinical characteristics, and psychosocial status were also explored. | Longitudinal survey study of patients receiving capecitabine for gastrointestinal malignancy | Higher HL was predictive of better medication adherence in multiple regression analysis (b=2.43, p=0.03) |
| Joyce, 2020, USA[59] | To describe the relationship between health literacy and prostate cancer treatment regret in a cohort enriched with African American patients from both community based and academic settings. To identify patient-level predictors of health literacy and assess the relationship between health literacy, numeracy, and prostate-related knowledge. | Cross sectional survey of men with prostate cancer attending two centres | Those with lower HL were more likely to express treatment regret (p<0.05). |
| Kappa, 2017, USA[60] | To better understand the discharge needs of patients who underwent radical cystectomy | Retrospective notes review of patients who had undergone radical cystectomy at single centre | Compared to patients discharged home without, those who required discharge services had lower HL scores on bivariate analysis (11.9 vs 12.5, p=0.016), but not multivariable analysis. |
| Kim, 2001, USA[61] | To evaluate knowledge, level of satisfaction, and treatment preferences and intentions of men newly diagnosed with prostate cancer after participation in the CD-ROM shared decision making program; and the relationship between prostate cancer knowledge and health literacy. | Evaluation of CD-ROM shared decision-making aid by newly diagnosed patients at single centre | Participants’ prostate cancer knowledge scores significantly positively correlated with their HL scores in bivariate analysis (Pearson correlation r=0.65 p=0.0001) |
| Koay, 2013, Australia[23] | To determine the prevalence of poor health literacy in people with lung or head and neck cancer using the Shortened-TOFHLA (S-TOFHLA) and HeLMS and to examine the association between health literacy levels and patients' socio-demographic factors and clinical characteristics, including distress. | Cross sectional survey of patients recently diagnosed with lung or head and neck cancer at single centre | Patients’ distress levels were not associated with HL using the S-TOFHLA measure (rho=0.034, p=0.744) but related to low scores in two domains of HeLMS measure ‘receptivity to health improvements’ (rho=0.276, p=0.007) and ‘communication with health professionals’ (rho=0.239, p=0.021). |
| Lee, 2018, South Korea[34] | To explore the level of health literacy, self‐care behaviours, and quality of life in older persons with lung cancer undergoing chemotherapy and to identify the factors associated with quality of life. | Cross sectional self-report questionnaire of older cancer patients (>60 years) who had undergone chemotherapy at single centre | General QOL and disease-related QOL were positively correlated with functional HL (r=0.361, p=0.001 and r=0.431, p<0.001 respectively). In regression analysis, HL was not found to be a predictor of general QOL, but was a predictor for the lung cancer subscale of QOL (β=0.191, p=0.045).  Functional HL was not significantly correlated with self-care behaviours (r=0.189, p=0.093) |
| Lillie, 2007, USA[35] | To examine the relationship of health literacy to several indicators of breast cancer patients’ information-processing styles and preferences. | Cross sectional self-administered questionnaire study of women post treatment for early stage disease at single centre | Women with higher HL answered significantly more questions correctly than those with lower HL (OR, 2.82; 95% CI, 1.31-6.07; p<0.01). Women with higher HL indicated a preference for more active participation in the decision to get the recurrence risk test [F(1,144) = 4.77, p=0.03], significant in unadjusted analysis only, and to use the results to make treatment decisions [F(1,144) = 18.56, p< 0.001]. than women with lower HL. |
| Lim, 2019, Australia[36] | To describe the levels of health literacy and experience of cancer care coordination in a cohort of Chinese migrant cancer patients and carers, and to examine factors associated with these. | Cross sectional survey of Chinese migrant cancer patients and carers from community support groups or identified through medical records | There was a positive correlation between HL and experience of cancer care coordination (first canonical correlation = 0.81, p<0.001) |
| Mahal, 2015, USA[37] | To determine whether Prostate Specific Antigen (PSA) anxiety or health literacy are associated with unproven use of early salvage ADT as initial management for PSA recurrence following radiotherapy. | Data from prospective, multi-site study of men with prostate cancer using records and questionnaires | In univariate analysis, men who had higher HL were significantly less likely to undergo salvage ADT compared with men who had lower HL (15.2% vs 26.3%, OR 0.50; 95% CI 0.29–0.88; p=0.016). This trended towards but was not significant on multivariate analysis (AOR 0.58,  95% CI 0.32–1.05; p=0.07) |
| Matsuyama, 2011, USA[38] | To examine and measure the associations between race, health literacy, and self-reported needs for information about disease, diagnostic tests, treatments, physical care, and psychosocial resources. | Cross sectional questionnaire study of patients undergoing cancer treatment at two sites | HL was significantly correlated with total (p<0.05), psychosocial and tangible information needs (both p<0.01) in bivariate analysis, but not in linear regression analysis. |
| McDougall, 2018, US [39] | To investigate rural disparities in financial hardship and its association with nonadherence to surveillance colonoscopy among a population based ethnically and geographically diverse sample of colorectal cancer survivors. | Cross sectional self-administered survey of patients identified through cancer registry, with oversampling of Hispanic and rural patients | Low HL was independently associated with financial hardship in a multivariate adjusted model (OR 5.41, 95% CI 1.45–20.1, p<0.05) There was no reported association between HL and adherence to surveillance colonoscopy. |
| McDougall, 2019, USA[40] | To investigate associations between socioeconomic factors, including annual household income, health literacy, and insurance and patient-reported HRQOL in a population-based, ethnically and geographically diverse sample of CRC survivors | Cross sectional self-administered survey of patients identified through cancer registry, with oversampling of Hispanic and rural patients | Low HL was associated with an average 4.24-point (95% CI 1.70–6.77) higher pain interference score, 3.28-point higher sleep disturbance score (95% CI 0.71–5.68) and 2.89-point (95% CI 0.42–5.37) higher depression score in multivariate analysis (all significant at p<0.05) |
| Mohan, 2009, USA[41] | To survey newly diagnosed patients about their anticipation of survival with and without treatment. | Cross sectional self-administered survey of newly diagnosed men with localised prostate cancer from single centre | PDLO and PILT were not related to health literacy |
| Nakata, 2020, Germany[62] | To investigate distribution and change of need for psycho-oncological care over the course of a breast cancer treatment, to investigate individual determinants for reporting a need for psycho-oncological support with special emphasis on individual health literacy, fear of progression, and mental disorders, to clarify whether there are positive or negative associations between health literacy levels and the need for psycho-oncological care. | Part of a prospective, longitudinal, multicentre cohort study, assessment during inpatient stay and subsequent postal survey | Patients with inadequate HL were significantly more likely to develop a need for psychological support than those with sufficient HL (OR = 1.97; 95%-Cl = 1.26–3.08, p=0.003) in multiple logistic regression analysis |
| Nilsen, 2019, USA[24] | To determine the prevalence and predictors of inadequate health literacy and evaluate the association between health literacy and QOL in HNC survivors. | Retrospective analysis of routine data from survivorship clinic questionnaire | Inadequate HL was significantly associated with lower clinically meaningful social QOL scores compared to high HL (mean score 65.9 v. 79.7, p=0.013) but not physical QOL scores (69.6 v. 77.9, p=0.13) when adjusting for covariates |
| Ousseine, 2020, France[42] | To study medico-social follow-up and its associated determinants with a focus on HL among cancer survivors, using a national representative sample of cancer survivors in France 5 years after diagnosis | Analysis of survey data from patients registered on disease database | Low HL increased the likelihood of follow up by GP (OR 1.52, 95% CI 1.26-1.84) and contact with social worker (OR 1.40, 95% CI 1.11-1.77) in multivariable analysis. Those with limited HL were more likely to report anxiety, depression, fatigue (57.2% v 43.6% fatigue score ≥40) and sequelae following treatment (all p<0.001). |
| Ozkaraman, 2019, Turkey[43] | To assess the effect of health literacy on cancer patients' self-efficacy and quality of life | Cross sectional questionnaire study of patients attending medical oncology clinics at single centre | There was a significant positive relationship between HL and the general health subscale score (r=0.199, p=0.036) and a significant negative relationship with the symptom subscale score (r=-0.334, p=<0.001)  There was no significant relationship between HL and self-efficacy. |
| Parker, 2020, USA[63] | To describe the relationships between patients' health literacy, demographic factors, and cancer treatment characteristics with chemotherapy knowledge among women with breast cancer undergoing intravenous chemotherapy. | Cross sectional survey of women with early stage disease at single centre | HL was significantly associated with chemotherapy knowledge in univariate analysis (p<0.05) |
| Plummer, 2017, Australia[44] | To examine the relationship between health literacy and physical activity in women who had completed treatment for breast cancer. | Cross sectional telephone survey of women who had completed treatment for breast cancer attending a single centre | Moderate positive correlations were found between physical activity and functional (r = 0.474, p<0.01) and interactive HL (r = 0.443, p<0.01). A weak but significant positive correlation between physical activity and critical HL was also found (r = 0.358, p<0.05). In stepwise regression analyses, functional health literacy (p=0.004) and “having sufficient information to manage health” (p=0.001) were significant predictors. |
| Polite, 2019, USA[45] | To test the effectiveness of an interactive health communication tool to improve the knowledge, self-efficacy, attitudes, willingness, and receptivity of patients with cancer regarding therapeutic cancer clinical trials, to evaluate the feasibility and acceptability of multimedia technology for patient-reported outcomes assessment and patient education in an oncology clinic, and to evaluate the impact of the health communication tool on clinical trial discussion and enrolment. | Single centre study testing effectiveness of a multimedia cancer clinical trials intervention | Patients with high HL demonstrated an increased willingness to take part in a clinical trial if one was offered (p=0.049) but were not more likely to have discussed clinical trials with their physician.  There was no significant difference in decision-making preferences between those with high and those with low HL. |
| Post, 2020, USA[46] | To describe the relationship between sociodemographic factors, survivorship variables, and patient engagement in breast cancer survivors and to explore how variations in these variables might contribute to patient engagement in breast cancer survivors. | Cross-sectional web-based self-report national survey of women with early stage breast cancer | HL positively correlated with patient engagement variables (knowing participation in change and patient activation) in bivariate analysis only (r=0.631, p≤0.001 and r=0.270, p≤0.001 respectively). |
| Rust, 2015, USA[72] | To assess the impact of a skills intervention on medication adherence, health literacy, and self-efficacy among African-American breast cancer survivors | Randomised controlled trial of medication adherence skills training, patients recruited from community support organisations | Higher HL was related to higher levels of medication adherence (B=0.582, 95% CI [28.42, 41.73], r=0.29, p=0.044) and self-efficacy for medication use (B=0.834,95% CI [12.29, 30.50], r=0.32, p=0.027) in linear regression analysis |
| Scarpato, 2016, USA[64] | To investigate the relationship between health literacy and surgical outcomes | Retrospective database review of patients who had undergone radical cystectomy at single centre | Lower HL was significantly associated with developing a minor complication in multivariable logistic regression analysis (OR 0.90, 95% CI 0.83-0.97, p<0.05). HL was not significantly associated with time to first ED visit or readmission. |
| Smith, 2020, Australia[65] | To explore levels and correlates of clinical trial knowledge and attitudes in Vietnamese- versus Anglo-Australian cancer patients. | Cross-sectional self-report questionnaire study | Higher HL was associated with better trials knowledge in multivariable regression analysis (B=0.21; 95% CI 0.01-0.40, p=0.04). Lower HL was associated with poorer trials attitudes in univariate analysis only (B=2.02, 95% CI 0.92–3.13, p<0.001) |
| Song, 2012, USA[66] | To examine whether health literacy is related to HRQOL among men with newly diagnosed clinically localised prostate cancer | Cross sectional multi-centre study using structured questionnaires delivered in person and records review | HL was significantly related to physical wellbeing in the crude model (p<0.0001) but not when controlling for covariates. HL was significantly associated with mental wellbeing in the crude model and remained significant in the adjusted models (B=1.86, p=0.0394) |
| Tagai, 2020, USA[47] | To identify medical and social determinants associated with self-efficacy for re-entry, perceived quality of interactions with medical providers, and practical concerns among individuals in their first year post-treatment for localised prostate cancer | Cross sectional questionnaire study at four centres using baseline data from earlier randomised controlled trial | Those with higher HL had greater self-efficacy for re-entry in multivariable regression analysis (p<0.001) and reported better interactions with their providers (p<0.05). Those with higher HL reported fewer practical concerns in multivariable analysis (β=−0.11, p<0.05) |
| Turkoglu, 2019, Turkey[73] | To investigate the relationship between bladder cancer patients' compliance with the cystoscopic follow-up and treatment protocol and HL. | Cross sectional study of patients who had undergone surgery for bladder cancer at a single centre | The treatment continuity rate was significantly higher in the adequate HL group than in the inadequate HL group (80.50% v 56.50% respectively, p=0.008) in bivariate analysis. |
| Watson, 2020, USA[67] | To gain a realistic understanding of gynaecologic oncology patients’ use of oral anticancer medications through both a quantitative and a qualitative exploration of adherence. | Cross sectional questionnaire and selected interviews with women receiving oral anticancer treatment | There was no significant difference in HL between adherent and non-adherent groups |
| Winton, 2016, USA[25] | To examine factors that affect use of lumpectomy, mastectomy alone, and reconstruction after mastectomy in an underinsured population. | Cross sectional study of women with breast cancer attending a single centre | Adequate HL was associated with a greater likelihood of breast reconstruction but did not reach statistical significance in multivariate analysis (OR 3.13; 95% CI, 0.95–10.30, p=0.06). |
| Wolpin, 2016, USA[74] | Observational study, using an eye tracker device, of how men with newly diagnosed localized prostate cancer visually engaged with an on-screen infographic depicting risk information in the Personal Patient Profile–Prostate. | Single centre observational study of eye-tracking data whilst participants used a web-based treatment decision intervention | Eye tracking patterns differed between levels of HL. Participants with lower HL spent more time on the Years To Live text and on the infographic than those with high HL |
| Xia, 2019, China[48] | To evaluate the association between HL and QOL in a sample of cancer survivors from the Shanghai Cancer Rehabilitation Club using three brief screening questions | Cross sectional survey of patients with cancer attending rehabilitation club | Lower HL was significantly associated with poorer quality of life in logistic regression analysis (adjusted OR 2.81; 95% CI 1.94 to 4.06; p<0.001) |
| Yen, 2020, USA[68] | To determine if Option Grid or Picture Option Grid encounter conversation aids increased observed SDM compared with usual care when used in surgical visits for early-stage breast cancer; to determine if observed SDM varied by patient characteristics indicative of being disadvantaged, and to determine if observed SDM and patient reported SDM overall correlate, while controlling for intervention and patient characteristics. | Secondary analysis of data collected from randomised controlled trial at four centres | Observed shared decision-making scores did not significantly differ between patients with inadequate and adequate HL (crude mean score 50.9 v 52.9 t=0.67, p=0.50) |
