# Supplementary material for: The role of health literacy in cancer care: A mixed studies systematic review
Source: PLoS One. 2021 Nov 12;16(11):e0259815. doi: 10.1371/journal.pone.0259815 (PMC8589210; doi:10.1371/journal.pone.0259815)
Supplement: S1 File — (DOCX) [file pone.0259815.s002.docx]

**S1 File: Full search strategy**

Search carried out in Ovid MEDLINE 15/01/2021:

1. exp health literacy/
2. health competence.mp.
3. health literacy.mp.
4. exp Neoplasms/
5. cancer*.tw.
6. malignanc*.tw.
7. neoplasm*.tw.
8. tumo?r*.tw.
9. carcinoma*.tw.
10. 4 or 5 or 6 or 7 or 8 or 9
11. 1 or 2 or 3
12. 10 and 11

Search carried out in Ovid EMBASE 15/01/2021:

1. exp health literacy/
2. health competence.mp.
3. health literacy.mp.
4. exp Neoplasms/
5. cancer*.tw.
6. malignanc*.tw.
7. neoplasm*.tw.
8. tumo?r*.tw.
9. carcinoma*.tw.
10. 4 or 5 or 6 or 7 or 8 or 9
11. 1 or 2 or 3
12. 10 and 11

Search carried out in EBSCO PsycINFO 15/01/2021:

1. DE "health literacy"
2. "health competence"
3. "health literacy"
4. DE "Neoplasms" OR DE "Breast Neoplasms" OR DE "Endocrine Neoplasms" OR DE "Leukemias" OR DE "Melanoma" OR DE "Metastasis" OR DE "Nervous System Neoplasms" OR DE "Terminal Cancer"
5. TI cancer*
6. AB cancer*
7. TI malignanc*
8. AB malignanc*
9. TI neoplasm*
10. AB neoplasm*
11. TI tumo?r*
12. AB tumo?r*
13. TI carcinoma*
14. AB carcinoma*
15. S4 OR S5 OR S6 OR S7 OR S8 OR S9 OR S10 OR S11 OR S12 OR S13 OR S14
16. S1 OR S2 OR S3
17. S15 AND S16

Search carried out in EBSCO CINAHL 15/01/2021:

1. MH "health literacy"
2. "health competence"
3. "health literacy"
4. MH "neoplasms+"
5. TI cancer*
6. AB cancer*
7. TI malignanc*
8. AB malignanc*
9. TI neoplasm*
10. AB neoplasm*
11. TI tumo?r*
12. AB tumo?r*
13. TI carcinoma*
14. AB carcinoma*
15. S4 OR S5 OR S6 OR S7 OR S8 OR S9 OR S10 OR S11 OR S12 OR S13 OR S14
16. S1 OR S2 OR S3
17. S15 AND S16
