# Supplementary material for: The role of health literacy in cancer care: A mixed studies systematic review
Source: PLoS One. 2021 Nov 12;16(11):e0259815. doi: 10.1371/journal.pone.0259815 (PMC8589210; doi:10.1371/journal.pone.0259815)
Supplement: S2 File — (DOCX) [file pone.0259815.s003.docx]

The role of health literacy in cancer care: Protocol for a systematic review

# Background

Health literacy is a complex concept which has varied in definition and meaning according to discipline and over the years since its introduction in the 1970s (Okan et al., 2019, Sørensen et al., 2012, Friedman and Hoffman-Goetz, 2008). From initially referring to the ability to read and use medical information, definitions of health literacy now reflect developments in the understanding of not only the personal knowledge requirements, but also the skills, confidence and external influences which enable people to use this knowledge to take an active role in their own health (Okan et al., 2019). This is brought together in the 2015 World Health Organisation definition of health literacy as the personal characteristics and social resources needed for individuals and communities to access, understand, appraise and use information and services to make decisions about health. Health literacy includes the capacity to communicate, assert and enact these decisions (World Health Organization, 2015).

The changing definitions and understanding of the concept are reflected in the numerous tools designed to measure health literacy. The Health Literacy Tool Shed database of health literacy measures currently lists 191 tools, which range in complexity, time and method of administration, language and context (Health Literacy Tool Shed, 2020). The earlier, and most frequently used (Berkman et al., 2011b, Okan et al., 2019) tools to measure health literacy include the Rapid Estimate of Adult Literacy in Medicine (REALM) tool, a word recognition test developed to identify patients in the clinic with limited reading skills (Davis et al., 1991), and the Test of Functional Health Literacy in Adults (TOFHLA), developed to test both reading comprehension and numeracy (Parker et al., 1995). Perhaps the simplest measure is the Single Item Literacy Screener (SILS), which asks, “How often do you need to have someone help you when you read instructions, pamphlets, or other written material from your doctor or pharmacy?” to identify those who may need help with written information (Morris et al., 2006). More recent tools including the Health Literacy Questionnaire (HLQ) (Osborne et al., 2013) and European health literacy survey (HLS-EU-Q) (Sørensen et al., 2013) are multi-item self-report measures and have been developed using conceptual frameworks to reflect the more complex current understanding of health literacy.

In view of the evolving definitions, concepts and tools, the study of health literacy and comparisons over time can be challenging, however, there is clear evidence that limited health literacy can lead to poorer outcomes for patients and increased burden on emergency healthcare services (Berkman et al., 2011a, Berkman et al., 2011b). Encouragingly, health literacy appears to be a modifiable entity (Visscher et al., 2018, Okan et al., 2019), it also appears possible to mitigate the negative consequences of limited health literacy in practice, although further evidence to demonstrate this is needed (Berkman et al., 2011b).

As well as relationships with direct health outcomes, health literacy is an important prerequisite for participation in decision making (Bravo et al., 2015, Sørensen et al., 2012, Edwards et al., 2012), the process by which patients and clinicians work together to reach decisions about care by combining both the clinician’s expertise and the patient’s expertise in themselves as a subject (NHS England, no date). Shared decision making is a key component of Universal Personalised Care (NHS England, 2019) and its use is embedded throughout the NICE guidance. In terms of cancer care, this includes the many guidelines outlining the diagnosis and management recommendations for a range of malignancies under the heading of Patient-centred Care (National Institute for Health and Care Excellence (NICE), 2011).

Patients diagnosed with cancer may face many difficult decisions, including those around treatments such as the use of systemic anti-cancer therapy (including chemotherapy, immunotherapy and targeted therapies), as well as radiotherapy and surgery, participation in clinical trials, decisions about palliative care involvement and cardiopulmonary resuscitation, and, ultimately, advance care planning and end of life decisions. Sometimes there is clear evidence that one option is superior to another, at other times there is equipoise, but any decision made should take into account the patient’s needs and preferences (National Institute for Health and Care Excellence (NICE), 2019).

In addition to taking an active role in decisions about their care, we also expect patients to read and digest the information about the proposed management, to understand a whole new language of health terminology with which they may be unfamiliar, ask them to provide consent for treatments and procedures, and turn up at the right place and the right time for their appointments. This all relies on a person having adequate health literacy and it is possible to see how problems may arise if this is not the case. A scoping review by Papadakos et al. (2018) looking at health literacy and cancer self-management behaviours suggests a link between limited health literacy and reduced uptake of prescribed chemotherapy, as well as increased information needs, supporting further exploration in this area.

The aim of this systematic review is therefore to further explore the wider impact of limited health literacy in patients with cancer.

# Review objectives

1. To identify which outcomes relate to limited health literacy in patients with cancer
2. To identify the prevalence of limited health literacy in patients with cancer
3. To identify what qualitative studies have (explicitly) explored the role of health literacy in patients to access, understand, appraise and use information and services to make decisions about health
4. To explore what interventions have been developed or tested to support patients with limited health literacy in this setting

# Methods

This protocol has been written in line with the PRISMA-P (preferred reporting items for systematic review and meta-analysis protocols) 2015 checklist. The review has been prospectively registered with the International Prospective Register for Systematic Reviews (PROSPERO), entry number…

## Eligibility

Searches will be carried out using the MeSH terms and keywords below order to identify all literature pertaining to health literacy in cancer. Search terms have been identified based on the PICOS tool and through review of the existing literature.

As we anticipate there to be a limited number of studies in this setting, we have not stipulated specific outcomes for study inclusion, though these are likely to include outcomes related to healthcare utilisation, information needs and patient reported outcomes.

|  | Inclusion | MeSH terms | Keywords |
| --- | --- | --- | --- |
| Population | Adult patients with cancer | Neoplasms | Cancer  Malignancy  Neoplasm  Tumour  Carcinoma |
| Intervention | All or no interventions included |  |  |
| Concept | Health literacy | Health literacy | Health literacy  Health competence |
| Outcomes | Outcomes including but not limited to healthcare utilisation, treatment decisions, patient reported outcomes |  |  |
| Studies | Not limited, both quantitative and qualitative studies to be included |  |  |

## Information sources

The following databases will be searched for relevant existing systematic reviews:

Cochrane database of systematic reviews

Database of abstracts of review of effectiveness (DARE)

The following electronic databases will be searched for relevant studies from inception to date of search:

MEDLINE

EMBASE

PsycINFO

CINAHL

Visual scanning of reference lists from included studies as well as citation searching of key papers will be undertaken.

A search the US National Library of Medicine Clinicaltrials.gov register will also be performed to identify any relevant ongoing and unpublished studies.

## Search strategy

A draft search strategy for use in Ovid MEDLINE is below:

1. exp health literacy/

2. health competence.mp.

3. literacy.mp.

4. exp Neoplasms/

5. cancer*.tw.

6. malignanc*.tw.

7. neoplasm*.tw.

8. tumo?r*.tw.

9. carcinoma*.tw.

10. 4 or 5 or 6 or 7 or 8 or 9

11. 1 or 2 or 3

12. 10 and 11

A pilot search of these terms was undertaken in Ovid MEDLINE on 11/10/19 and produced 1449 results. The results were crosschecked with previously identified articles to ensure accurate detection.

Subsequent refinement 17/10/19 and search carried out in Ovid MEDLINE:

1. exp health literacy/

2. health competence.mp.

3. health literacy.mp.

4. exp Neoplasms/

5. cancer*.tw.

6. malignanc*.tw.

7. neoplasm*.tw.

8. tumo?r*.tw.

9. carcinoma*.tw.

10. 4 or 5 or 6 or 7 or 8 or 9

11. 1 or 2 or 3

12. 10 and 11

Produced 961 hits.

# Study records

## Data management

Citations will be managed through Endnote X9 and Microsoft Excel.

## Study selection

Screening of each title and abstract will be undertaken by two independent reviewers, with a preference for inclusion if there are disagreements at this stage.

Following the initial screening process, full texts of the remaining studies will be reviewed for eligibility by two authors according to the following inclusion and exclusion criteria:

| Objective | Inclusion | Exclusion (all objectives) |
| --- | --- | --- |
| To identify which outcomes relate to limited health literacy in patients with cancer | - Patients aged >18 years - Malignancy of any site - Identifiable outcome - Health literacy measured with validated measure, not assumed e.g. on education or socioeconomic status ** - English language only | - Studies involving only children/adolescents - Studies looking at health literacy of other parties e.g. caregivers, healthcare providers or populations without a diagnosis of malignancy - Studies where ‘health literacy’ refers to disease specific knowledge only - Non-English language studies - Case reports - Review papers - Conference proceedings - Opinion pieces/editorials/letters to editor - Dissertations/theses - Book chapters - Study protocols |
| To identify the prevalence of limited health literacy in patients with cancer | - Patients aged >18 years - Malignancy of any site - Health literacy measured with validated measure, not assumed e.g. on education or socioeconomic status** - Prevalence of limited or low health literacy stated - English language only |  |
| To identify what qualitative studies have (explicitly) explored the role of health literacy in patients to access, understand, appraise and use information and services to make decisions about health | - Patients aged >18 years - Malignancy of any site - English language only - Qualitative studies where the role of health literacy in patients accessing, understanding, appraising and using information and services to make decisions about health is explored - Health literacy not necessarily measured - English language |  |
| To explore what interventions have been developed or tested to support patients with limited health literacy in this setting | - Patients aged >18 years - Malignancy of any site - English language only   EITHER   - Studies describing the development of an intervention to support patients with limited health literacy   OR   - Studies testing an intervention to support patients with limited health literacy, in which case health literacy must be measured, not assumed e.g. on education or socioeconomic status ** |  |

** For the purpose of this review, we will include measures that have previously been validated, looking at general health literacy or general cancer health literacy, but not tumour site specific health literacy. Measures must be used in their validated form in their entirety, with no missing or additional questions or components unless the results of these are reported separately.

Any disagreements at this stage will be discussed between the two reviewers. If a consensus cannot be reached, then the review group will meet to discuss the case and ultimately reach an agreement.

## Data extraction

Data extraction will be carried out using a data extraction form designed for the review and agreed on by all reviewers prior to implementation. It will be piloted on the first five studies selected for inclusion through the selection process. Once finalised, one reviewer will carry out data extraction for the remaining studies, and 10% will be checked by a second reviewer for completeness.

Data will be collected as below:

1. Study characteristics (author, year, country study undertaken, setting, design, aims/objectives, inclusion/exclusion, recruitment procedure, health literacy measure used and how limited health literacy defined)
2. Population (age range, sex ratio, cancer site, stage, number of participants and number of eligible participants if mixed group, proportion limited health literacy according to measure used and by tumour site)
3. Intervention (details of type, time to administer, effect on any outcomes measured)
4. Outcomes (as reported in individual studies, measures used and effect of health literacy on these)
5. Qualitative methods used, data analysis procedure, key themes and findings, participant quotes

Attempts will be made to obtain data missing from corresponding authors for studies selected for inclusion.

## Quality assessment

Quality of study data will be assessed using the Mixed Methods Appraisal Tool (MMAT) (Hong et al., 2018).

Quality assessment will be carried out by two independent reviewers. The assessment of quality will be recorded but not used to exclude studies from the review.

Any disagreements will initially be discussed between the two reviewers, and ultimately with the rest of the research team as needed to reach a resolution.

# Data synthesis

Due to the inclusive nature of this review and in addition to differing study designs, we anticipate that a number of different measures and thresholds will have been used to identify participants with limited health literacy, and statistical pooling of data in unlikely to be possible. We therefore plan to undertake a narrative synthesis, drawing on guidance developed by Popay et al. (2006).

The initial stage will entail description of the data from each study, including details of the study setting and population, the health literacy measures used and how limited or low health literacy is defined in the study, the prevalence of low or limited health literacy and any reported associated outcomes. We will also describe any interventions that have been developed in terms of their format (e.g. a 20 minute video, a six session educational programme) and, for those that have been tested, note the effect on reported outcomes.

Studies will be grouped and tabulated based on the four review objectives: 1) to identify which outcomes relate to limited health literacy in patients with cancer, 2) to identify the prevalence of limited health literacy in patients with cancer, 3) to identify what qualitative studies have (explicitly) explored the role of health literacy in patients to access, understand, appraise and use information and services to make decisions about health and 4) to explore what interventions have been developed or tested to support patients with limited health literacy in this setting. We will then undertake a process of concept mapping, visually displaying the links between the key concepts identified from the studies.

Prevalence of limited health literacy will be presented as a range, taking into account and acknowledging the differences in measures used and definitions of what is considered to constitute limited health literacy.

Finally, we will reflect critically on the process as a means of assessing the robustness of the synthesis.

# Dissemination

Findings will be submitted for publication in a peer-reviewed journal.

# Funding

CH has received funding as part of a PhD studentship from the Robert White Legacy Fund. The funder has had no role in development of this protocol.

# References

BERKMAN, N. D., SHERIDAN, S. L., DONAHUE, K. E., HALPERN, D. J. & CROTTY, K. 2011a. Low health literacy and health outcomes: an updated systematic review. *Ann Intern Med,* 155**,** 97-107.

BERKMAN, N. D., SHERIDAN, S. L., DONAHUE, K. E., HALPERN, D. J., VIERA, A., CROTTY, K., HOLLAND, A., BRASURE, M., LOHR, K. N., HARDEN, E., TANT, E., WALLACE, I. & VISWANATHAN, M. 2011b. Health literacy interventions and outcomes: an updated systematic review. *Evid Rep Technol Assess (Full Rep)***,** 1-941.

BRAVO, P., EDWARDS, A., BARR, P. J., SCHOLL, I., ELWYN, G. & MCALLISTER, M. 2015. Conceptualising patient empowerment: a mixed methods study. *BMC Health Services Research,* 15.

DAVIS, T. C., CROUCH, M., LONG, S. W., JACKSON, R. H., BATES, P., GEORGE, R. B. & BAIRNSFATHER, L. E. 1991. Rapid assessment of literacy levels of adult primary care patients. *Family medicine,* 23**,** 433-435.

EDWARDS, M., WOOD, F., DAVIES, M. & EDWARDS, A. 2012. The development of health literacy in patients with a long-term health condition: the health literacy pathway model. *BMC Public Health,* 12**,** 130.

FRIEDMAN, D. B. & HOFFMAN-GOETZ, L. 2008. Literacy and health literacy as defined in cancer education research: A systematic review. *Health Education Journal,* 67**,** 285-304.

HEALTH LITERACY TOOL SHED. 2020. *Health Literacy Tool Shed - A database of health literacy measures* [Online]. Available: <http://healthliteracy.bu.edu/all> [Accessed 19/08/2020 2020].

HONG, Q. N., PLUYE, P., FÀBREGUES, S., BARTLETT, G., BOARDMAN, F., CARGO, M., DAGENAIS, P., GAGNON, M.-P., GRIFFITHS, F. & NICOLAU, B. 2018. Mixed methods appraisal tool (MMAT), version 2018. *Registration of copyright,* 1148552**,** 10.

MORRIS, N. S., MACLEAN, C. D., CHEW, L. D. & LITTENBERG, B. 2006. The Single Item Literacy Screener: evaluation of a brief instrument to identify limited reading ability. *BMC family practice,* 7**,** 21-21.

NATIONAL INSTITUTE FOR HEALTH AND CARE EXCELLENCE (NICE). 2011. Colorectal cancer: diagnosis and management. Available: <https://www.nice.org.uk/guidance/cg131> [Accessed 14/10/2019].

NATIONAL INSTITUTE FOR HEALTH AND CARE EXCELLENCE (NICE). 2019. *Shared decision making* [Online]. Available: <https://www.nice.org.uk/about/what-we-do/our-programmes/nice-guidance/nice-guidelines/shared-decision-making> [Accessed 14/10/2019 2019].

NHS ENGLAND 2019. Universal Personalised Care: Implementing the Comprehensive Model. <https://www.england.nhs.uk/publication/universal-personalised-care-implementing-the-comprehensive-model/>.

NHS ENGLAND, N. E. no date. *Shared decision making* [Online]. Available: <https://www.england.nhs.uk/shared-decision-making> [Accessed 14/10/2019 2019].

OKAN, O., BAUER, U., LEVIN-ZAMIR, D., PINHEIRO, P. & SØRENSEN, K. 2019. *International Handbook of Health Literacy : Research, practice and policy across the lifespan,* Bristol, Policy Press.

OSBORNE, R. H., BATTERHAM, R. W., ELSWORTH, G. R., HAWKINS, M. & BUCHBINDER, R. 2013. The grounded psychometric development and initial validation of the Health Literacy Questionnaire (HLQ). *BMC Public Health,* 13**,** 658.

PAPADAKOS, J. K., HASAN, S. M., BARNSLEY, J., BERTA, W., FAZELZAD, R., PAPADAKOS, C. J., GIULIANI, M. E. & HOWELL, D. 2018. Health literacy and cancer self-management behaviors: A scoping review. *Cancer*.

PARKER, R. M., BAKER, D. W., WILLIAMS, M. V. & NURSS, J. R. 1995. The test of functional health literacy in adults. *Journal of general internal medicine,* 10**,** 537-541.

POPAY, J., ROBERTS, H., SOWDEN, A., PETTICREW, M., ARAI, L., RODGERS, M., BRITTEN, N., ROEN, K. & DUFFY, S. 2006. Guidance on the conduct of narrative synthesis in systematic reviews. *A product from the ESRC methods programme Version,* 1**,** b92.

SØRENSEN, K., VAN DEN BROUCKE, S., FULLAM, J., DOYLE, G., PELIKAN, J., SLONSKA, Z. & BRAND, H. 2012. Health literacy and public health: A systematic review and integration of definitions and models. *BMC Public Health,* 12**,** 80.

SØRENSEN, K., VAN DEN BROUCKE, S., PELIKAN, J. M., FULLAM, J., DOYLE, G., SLONSKA, Z., KONDILIS, B., STOFFELS, V., OSBORNE, R. H. & BRAND, H. 2013. Measuring health literacy in populations: illuminating the design and development process of the European Health Literacy Survey Questionnaire (HLS-EU-Q). *BMC Public Health,* 13**,** 948.

VISSCHER, B. B., STEUNENBERG, B., HEIJMANS, M., HOFSTEDE, J. M., DEVILLÉ, W., VAN DER HEIDE, I. & RADEMAKERS, J. 2018. Evidence on the effectiveness of health literacy interventions in the EU: a systematic review. *BMC public health,* 18**,** 1414-1414.

WORLD HEALTH ORGANIZATION 2015. *Health literacy toolkit for low-and middle-income countries: A series of information sheets to empower communities and strengthen health systems*, WHO Regional Office for South-East Asia.
